# Parity-time symmetry enabled ultra-efficient nonlinear optical signal processing

**DOI:** 10.1186/s43593-024-00062-w

**Published:** 2024-04-04

**Authors:** Chanju Kim, Xinda Lu, Deming Kong, Nuo Chen, Yuntian Chen, Leif Katsuo Oxenløwe, Kresten Yvind, Xinliang Zhang, Lan Yang, Minhao Pu, Jing Xu

**Affiliations:** 1 School of Optical and Electronic Information, Huazhong University of Science and Technology, Luoyu Road 1037#, Wuhan, 430074 China; 2 DTU Electro, Department of Electrical and Photonics Engineering, Technical University of Denmark, Ørsteds Plads 343, Kongens Lyngby, 2800 Denmark; 3 Wuhan National Laboratory for Optoelectronics, Huazhong University of Science and Technology, Luoyu Road 1037#, Wuhan, 430074 China; 4 Optics Valley Laboratory, Hubei, 430074 China; 5 Department of Electrical and Systems Engineering, Washington University, St. Louis, MO 63130 USA

## Abstract

Nonlinear optical signal processing (NOSP) has the potential to significantly improve the throughput, flexibility, and cost-efficiency of optical communication networks by exploiting the intrinsically ultrafast optical nonlinear wave mixing. It can support digital signal processing speeds of up to terabits per second, far exceeding the line rate of the electronic counterpart. In NOSP, high-intensity light fields are used to generate nonlinear optical responses, which can be used to process optical signals. Great efforts have been devoted to developing new materials and structures for NOSP. However, one of the challenges in implementing NOSP is the requirement of high-intensity light fields, which is difficult to generate and maintain. This has been a major roadblock to realize practical NOSP systems for high-speed, high-capacity optical communications. Here, we propose using a parity-time (PT) symmetric microresonator system to significantly enhance the light intensity and support high-speed operation by relieving the bandwidth-efficiency limit imposed on conventional single resonator systems. The design concept is the co-existence of a PT symmetry broken regime for a narrow-linewidth pump wave and near-exceptional point operation for broadband signal and idler waves. This enables us to achieve a new NOSP system with two orders of magnitude improvement in efficiency compared to a single resonator. With a highly nonlinear AlGaAs-on-Insulator platform, we demonstrate an NOSP at a data rate approaching 40 gigabits per second with a record low pump power of one milliwatt. These findings pave the way for the development of fully chip-scale NOSP devices with pump light sources integrated together, potentially leading to a wide range of applications in optical communication networks and classical or quantum computation. The combination of PT symmetry and NOSP may also open up opportunities for amplification, detection, and sensing, where response speed and efficiency are equally important.

**Supplementary Information:**

The online version contains supplementary material available at 10.1186/s43593-024-00062-w.

Optical signal processing enables the processing of an optical data stream without converting it to an electrical signal. Nonlinear optical signal processing (NOSP), leveraging ultrafast optical nonlinearity resulting from the anharmonic electron response, allows for unprecedented signal processing speed and potentially lower energy consumption compared to electronic signal processing due to its transparency to data format and rate as well as the multi-channel processing capability [1–4]. Various applications have been explored, such as ultrafast optical switching [5, 6], wavelength conversion [7–15], multicasting [16], amplification [17, 18], demultiplexing [19, 20], regeneration [21–23], all-optical logic gates [24], and spectral efficient bandwidth allocation [25] in fiber-optic networks. However, NOSP is mainly explored in the transport network layer, and recent optical computing relies on linear optical processing [26, 27] due to weak optical nonlinearities in practical NOSP applications [28, 29]. New materials [5, 7–10, 16–18] and structures [11–13, 19] have been developed. One way to enhance nonlinear effects is to use microresonators with a high-quality factor (*Q*) [30, 31], which offer high nonlinear efficiency due to enhanced intracavity photon density and open new avenues for a wide range of nonlinear applications [32–34]. However, the enhanced nonlinear light-matter interaction comes at the cost of the response speed. By Fourier reciprocity [35], the resonance linewidth $\Delta \omega$—inversely related to the *Q*—causes temporal mixing of information (intersymbol interference) if the time interval between adjacent optical pulses becomes less than $\Delta t\sim 2\pi /\Delta \omega$. As a result, there is a fundamental tradeoff between the high-*Q* cavity enhanced nonlinearity and the maximum signal bandwidth $B$ (proportional to the data rate *D*, i.e., $1/\Delta t$) supported by a resonator*.* While various schemes have been investigated to overcome this bandwidth-efficiency limit [36–38], they often face challenges on structural complexity, footprint, limited performance, or are focused on quantum applications. Although strong nonlinear effects can also be achieved by increasing the interaction lengths of the nonlinear medium [17, 18], it is limited by a large device footprint, reduced phase-matching bandwidth, and demanding fabrication tolerance.

The recent advent of parity-time (PT) symmetry in optical resonant systems [39–42] suggests a new degree of freedom to control the flow of light in microcavities via gain and loss. By manipulating the PT symmetry, researchers have been able to demonstrate a range of unprecedented applications in lasers [43–45], microwave photonics [46], optical frequency combs [47], optical isolators [48], etc. In this work, we take advantage of PT symmetry to manipulate the linewidth of a coupled resonator system. Instead of having the same linewidth for every longitudinal resonance in a single cavity, the linewidths of the coupled cavities are manipulated over different longitudinal modes via utilizing the concept of exceptional point (EP) and spontaneous PT-symmetry breaking in different spectral windows simultaneously in the same structure. The intensity enhancements, which are directly related to the linewidth property of the coupled system and play an important role in FWM conversion efficiency, are manipulated in the frequency domain accordingly. Consequently, the property of the FWM conversion efficiency of the system is modified, enabling the breaking of the bandwidth-efficiency limit with two orders of magnitude improvement in the nonlinear efficiency compared to a single cavity system. Additionally, we demonstrate a high-speed and power-efficient wavelength conversion process based on AlGaAs-on-Insulator (AlGaAsOI) platform and verify its unique advantages in NOSP system performance, showing a record low pump power of 1 mW at a data rate of 38 Gbit/s with ultra-low power-penalty operation (< 1 dB). Our device also features a small footprint (about 0.01 mm^2^) and a broad wavelength conversion bandwidth (> 170 nm).

## PT symmetry-enabled linewidth manipulation

**Fig. 1:**
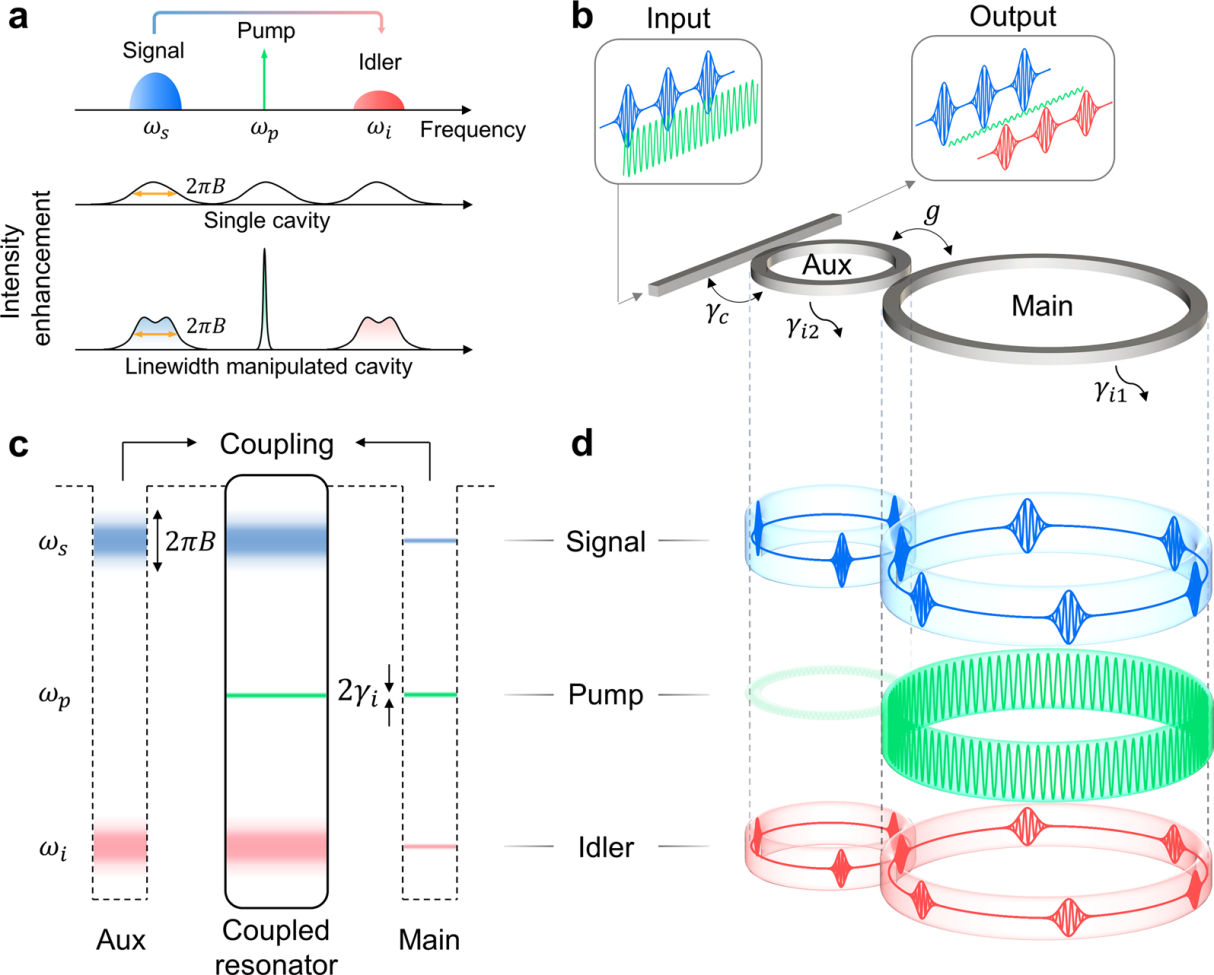
Parity-time (PT) symmetry-based manipulation of linewidth and intensity enhancement. **a** Upper: schematic of the wavelength conversion, where a strong continuous-wave (CW) pump light is applied to convert a high-speed optical data stream carried by a signal wave to an idler wave. Middle and bottom: illustrations of intensity enhancement spectrum of four-wave mixing (FWM) process used for wavelength conversion operation in a single cavity (middle) and linewidth manipulated cavity (bottom) designed for the same signal bandwidth *B*, respectively. **b**, **c** Schematic diagram and energy ladder of the PT-symmetric coupled microresonators, respectively. **d** Schematic diagram of the intracavity field distribution of signal, pump, and idler wave indicated by blue, green, and red colored shades, respectively. The signal and idler wave pulses in the cavities depict high-speed data-encoded signal and idler waves; in reality, the pulse durations are longer than the cavity roundtrip time

Among various types of nonlinear processes (e.g., second-order χ^(2)^ and third-order χ^(3)^ susceptibility, stimulated Raman scattering, stimulated Brillouin scattering), we employ degenerate four-wave mixing (FWM) based on χ^(3)^ for the NOSP. The degenerate FWM allows a flexible configuration of the pump wavelength, broadband operation with dispersion engineering [49], and benefits significantly from the cavity enhancement. The underlying principle of the linewidth manipulation in our PT symmetry system is illustrated in Fig. 1. It shows wavelength conversion based on cavity-enhanced FWM, which is of particular interests to overcoming wavelength contention at optical communication network nodes [14]. A broadband data stream carried by a signal wave (centered at round frequency ${\omega }_{s}$, blue) is converted to an idler wave (centered at ${\omega }_{i}$, red) mediated by a strong narrow-band continuous-wave (CW) pump [25] (centered at ${\omega }_{p}$, green), satisfying ${2\omega }_{p}={\omega }_{s}+{\omega }_{i}$ (Fig. 1a, upper panel). The conversion efficiency ($\eta$) is defined as the power ratio of the generated idler wave to the input signal wave. An important parameter related to $\eta$ is intensity enhancement factors $F$, which is defined as the ratio of intracavity mode intensity to the input field intensity. In general, $\eta$ scales proportional to ${F}_{p}^{2}{F}_{s}{F}_{i}$ [11], where ${F}_{p,s,i}$ are $F$ at ${\omega }_{p,s,i}$. It can be shown that $F$ is inversely proportional to the resonance linewidth $\Delta \omega$ in the limit of $\Delta \omega \gg {\gamma }_{i}$. In case of critical coupling ($\Delta \omega =2{\gamma }_{i}$, ${\gamma }_{i}$ is the intrinsic decay of the cavity), $F$ reaches maximum intensity enhancement ${F}_{{\text{max}}}$ (see Additional file 1: Eq. (S18), S4). To enable NOSP, the linewidth of the signal/idler resonances must be no smaller than $2\pi B$. In a single cavity, all the resonances share the same linewidth (Fig. 1a, middle panel). Therefore, ${F}_{p}$ scales inversely proportional to $B$. On the other hand, we notice that the pump light is a narrow-band wave and ${F}_{p}$ can be drastically increased in a linewidth-manipulated cavity (Fig. 1a, bottom panel). The linewidth of the pump resonance is selectively reduced to achieve ${F}_{\text{max}}$, while the linewidth of the signal and idler resonances is kept unchanged to facilitate high-speed operation. As a result, $\eta$ can be improved by a factor of ${\left({F}_{\tt{max}}/{F}_{B}\right)}^{2}$, where ${F}_{B}$ is defined as $F$ at $\Delta \omega =2\pi B$.

In this work, we propose a dual coupled microresonator system for the required linewidth manipulation, as shown in Fig. 1b. The coupled resonator device consists of a main cavity (intrinsic decay ${\gamma }_{i1}$ and cavity length ${L}_{1}$), an auxiliary cavity (intrinsic decay ${\gamma }_{i2}$ and cavity length ${L}_{2}$) with an intercavity coupling rate $g$. The auxiliary cavity is coupled to a bus waveguide with coupling decay ${\gamma }_{c}$. Figure 1c illustrates the energy ladder of the coupled system where the relation between two cavity lengths are set as ${L}_{1}=2{L}_{2}$. The main and auxiliary cavity resonances are aligned at ${\omega }_{s}$ and ${\omega }_{i}$ while the resonance at ${\omega }_{p}$ is only supported by the main cavity. A passive PT system is formed in the spatial domain by adding loss to the auxiliary cavity via the bus waveguide. When ${\gamma }_{c}\gg {\gamma }_{i1}\approx {\gamma }_{i2}$, the main cavity functions as an effective low-loss resonator while the auxiliary cavity—together with the bus waveguide, plays the role of an effective high-loss resonator.

With the condition of ${\gamma }_{c}\approx 4g$, the loss contrast between the two effective resonators at ${\omega }_{s}$ and ${\omega }_{i}$ are comparable to $4g$ (Additional file 1: S1), bringing the system to a near-EP condition. In the PT-symmetric regime (alternatively called split-frequency regime), light circulates in both cavities, causing the loss of light in the main cavity to increase due to the auxiliary resonator (effective high-loss resonator), which approaches its maximum near the EP condition. The interchange of the loss enables the existence of broadband (high-speed) signal and idler waves in the main cavity. On the other hand, the distinct operation of ${\omega }_{p}$ compared to ${\omega }_{s}$ and ${\omega }_{i}$ is constructed by taking the concept of PT-symmetry breaking. The destructive interference at ${\omega }_{p}$ leads to significant additional loss of the pump wave in the auxiliary cavity, allowing the revival of cavity enhancement in the main cavity by shifting the working condition to the PT-symmetry broken regime [45] (alternatively called split-dissipation regime). A more rigorous analysis can be found by transfer matrix method, i.e., the destructive interference at ${\omega }_{p}$ in the auxiliary cavity as well as the coupling between two cavities establish a quasi-critical coupling condition for the main resonator at pump frequency (Additional file 1: S3). The critical coupling condition of the pump light is defined as the extinction of the pump wave at the output port of the bus waveguide. In both pictures, the pump wave is highly localized in the resonator with effective low loss, i.e., the main cavity, ensuring great intensity enhancement of the pump wave. The intracavity fields (Fig. 1d) further elucidate the spatio-spectral resonator mode distributions of the alternating resonances characterized by broad and narrow linewidths. The signal and idler waves are evenly distributed between both cavities as the corresponding resonances are in the near EP of the PT-symmetric regime. In contrast, the pump wave is primarily confined in the main cavity attributed to the PT-symmetry breaking/quasi-critical coupling (see Additional file 1: S8 for finite element simulation). Therefore, the FWM process occurs predominantly in the main cavity, which is largely influenced by the localized distribution of the pump wave. In summary, the coupled resonator system is passive and that pump mode is operated in the split dissipation regime to maximize the power enhancement (conversion efficiency) in the main resonator whereas the signal and idler modes are operated in the split frequency regime of the coupled system (but close to EP) to maximize the supported signal bandwidth of the coupled resonator system.

## Synthetic linewidth formulation

**Fig. 2:**
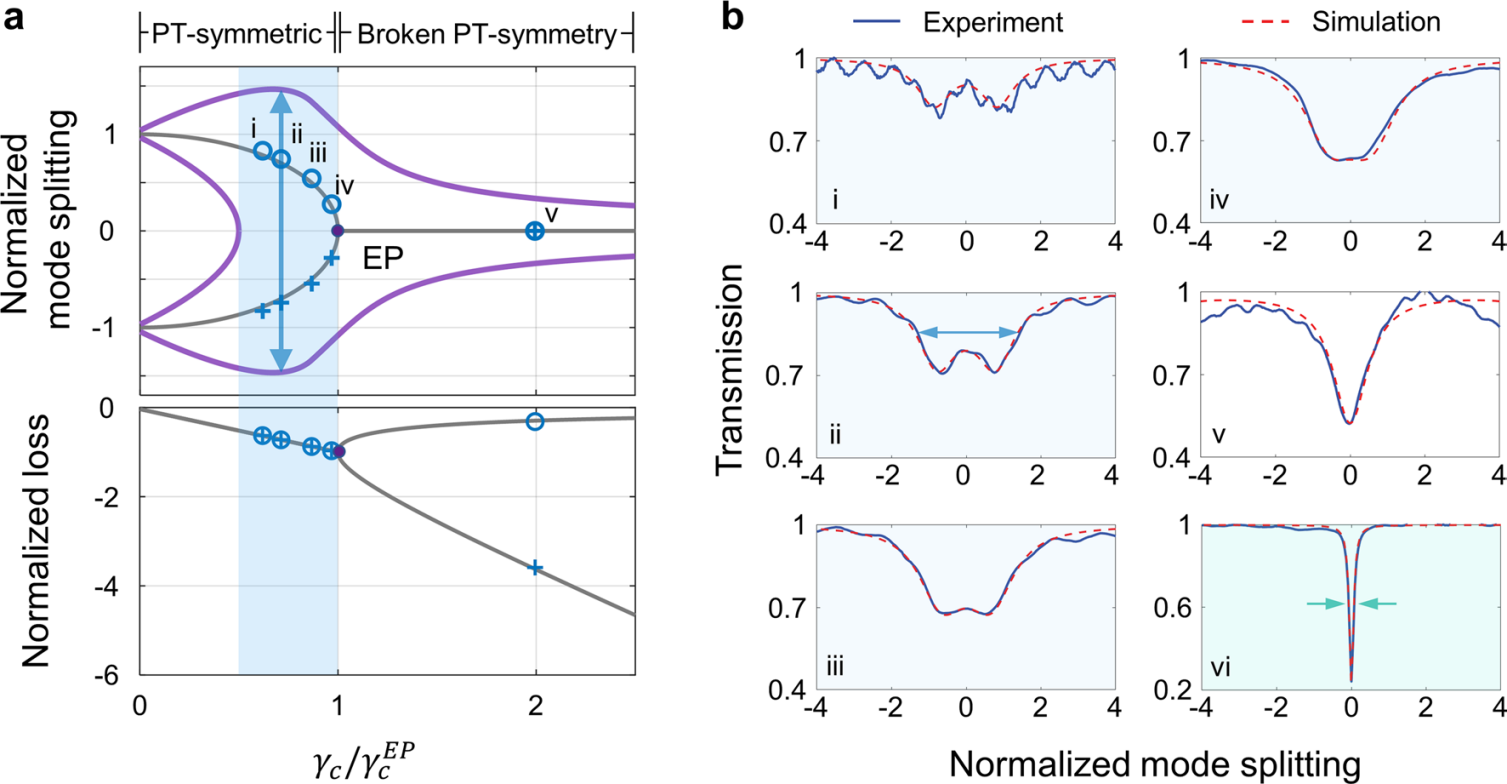
PT symmetry features of the dual coupled microresonator. **a** Evolution of the normalized real (mode splitting) and imaginary (loss) components of the eigenvalues of the passive PT symmetry system as a function of ${\gamma }_{c}$ (normalized by ${\gamma }_{c}$ at the EP, i.e.,${\gamma }_{c}^{{\text{EP}}}$). Purple solid lines plot the relative frequencies at which the normalized transmission becomes 1/2 at a given${\gamma }_{c}$. The blue-shaded area represents the preferred operating region of the signal and idler light. **b** Transmission spectra of the systems. The blue solid lines represent the measured transmission spectra, while the red dashed lines represent their curve fittings for devices operating at different coupling conditions. Panel (i) to (v) correspond to the blue data points shown in a from left to right. The ripples observed in the spectra are typical Fabry–Perot resonances resulting from the end facet reflections. Note that the transmission spectrum in each panel yields a total of four data points in (**a**)—mode splitting and loss corresponding to ${\omega }_{\pm }$, at different${\gamma }_{c}$. A three-dimensional plot showing the evolution of the transmission spectrum is provided in Additional file 1: S3. For comparison, panel (vi) shows the transmission spectrum near pump resonance where the horizontal axis means relative frequency with respect to ${\omega }_{p}$. Analysis of the pump operation from PT aspect as well as TMM are given in Additional file 1: S3. The simulations (red dashed lines) for the curve fitting are carried out based on the transfer matrix model (Additional file 1: S2). More details for data extraction are given in Methods

The principle of the linewidth manipulation can be further understood from the evolution of a synthetic linewidth of the coupled system, which is the key design parameter in the PT-symmetry-based NOSP system. The synthetic linewidth is closely related to the eigenfrequencies (${\omega }_{\pm }$) of the coupled ring resonator. According to the coupled mode theory, ${\omega }_{\pm }$ are complex values due to the characteristics of passive PT-symmetric systems [45] (Additional file 1: S1). The typical theoretical evolution of Re $({\omega }_{\pm })$ and Im $({\omega }_{\pm })$ for the eigenmodes are plotted as a function of ${\gamma }_{c}$ (normalized by ${\gamma }_{c}$ at the EP, i.e. ${\gamma }_{c}^{{\text{EP}}}$), which is indicated as gray lines in Fig. 2a. The synthetic linewidth is defined as the 3-dB bandwidth of the normalized transmission spectrum, which reflects the synthesized resonance of the two eigenmodes of the coupled PT symmetry system. As shown in Fig. 2a, the purple lines indicate the relative frequencies at which the normalized transmission becomes 1/2 at a given ${\gamma }_{c}$ (a detailed 3D evolution of the transmission spectrum is given in Additional file 1: S3). Therefore, the spectral distance between purple lines determines the synthetic linewidth (blue arrow). In the PT-symmetric regime near the EP, the distance between the central frequency of the two eigenmodes (${\text{Re}}({\omega }_{+})-{\text{Re}}({\omega }_{-})$) and the linewidths of the eigenmodes ($2{\text{Im}}\left({\omega }_{+}\right)$, or equivalently $2{\text{Im}}\left({\omega }_{-}\right)$) both contribute to the synthetic linewidth. In the PT-symmetry broken regime, where the two eigenmodes coalesce (${\text{Re}}\left({\omega }_{+}\right)-{\text{Re}}\left({\omega }_{-}\right)=0$), the synthetic linewidth of the system is determined by the linewidth of the low-loss eigenmode ($2{\text{Im}}({\omega }_{+})$). Therefore, the synthetic linewidth shows the combined feature of both the real and imaginary parts of ${\omega }_{\pm }$, approximated well by ${\Delta \omega }_{{\text{syn}}}={\text{Re}}({\omega }_{+})-{\text{Re}}({\omega }_{-})+2{\text{Im}}({\omega }_{+})$ at the regime close to the EP as well as the broken PT-symmetry regime (see more details in Additional file 1: S6). Note that the PT-symmetric regime far from the EP is particularly detrimental to the NOSP operation as the excessive mode splitting results in severe signal distortion. Also, the synthetic linewidth narrows when moving into the PT-symmetry broken regime. It is not favorable for our purposes of broadening the signal and idler resonances. However, this regime is advantageous for achieving maximum intensity enhancement for the pump operation.

Experimental validation has been carried out by fabricating a series of integrated coupled resonators that cover a broad range of coupling conditions. AlGaAs-on-insulator (AlGaAsOI) platform is used (fabrication details are given in Methods). The experimental transmission spectra near ${\omega }_{s,i}$ with different ${\gamma }_{c}$ are provided in Fig. 2b(i–v), where panels (i) to (v) correspond to the data points in Fig. 2a from left to right (see Methods for detailed data extraction and normalization). It is clear that a broad synthetic linewidth is obtained by merging two eigenmodes at a PT-symmetric regime near the EP (case i–iv, blue-shaded area). When ${\gamma }_{c}>{\gamma }_{c}^{{\text{EP}}}$, synthetic linewidth gets narrower [Fig. 2b(v)]. In the extreme case, an extremely narrow linewidth can be achieved, which facilitate pump operation. In Fig. 2b (vi), the transmission spectrum with sharply narrowed linewidth near ${\omega }_{p}$ is shown. A possible analysis from PT-symmetry breaking point of view is given in Additional file 1: S3. From the transfer matrix method analysis, the extremely narrow linewidth and near-zero transmittance at a resonant frequency confirms the quasi-critical coupling condition for the pump waves. Although this is consistent with the fact that intense pump light is accumulated in the main cavity, the phenomenon is counterintuitive from the viewpoint that pump frequency is not resonant in the auxiliary cavity. Further analyses show that the quasi-critical coupling condition can be achieved (${F}_{p}$ approaching ${F}_{\text{max}}$) over a wide range of synthetic signal/idler linewidths (Additional file 1: S3, S7).

The synthetic linewidth suitable for both signal/idler and pump waves promote the high-speed FWM process with enhanced conversion efficiency. The novel configuration is at the core of our realization toward an extremely low-power on-chip nonlinear signal processing photonic device. Relevant theoretical details are provided in Additional file 1: S1 to S5.

## Breaking bandwidth-efficiency limit using coupled AlGaAsOI microresonators

**Fig. 3:**
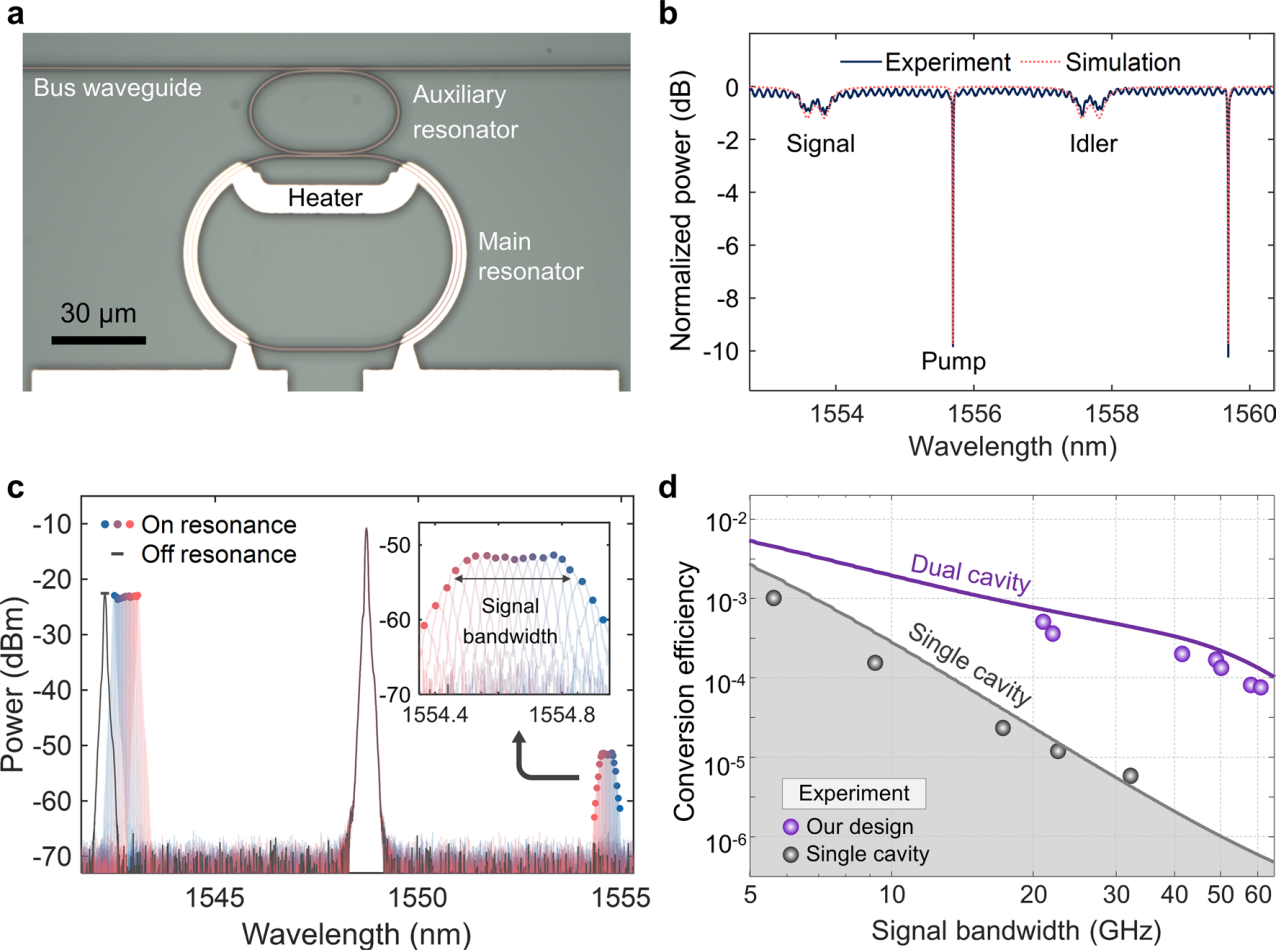
FWM in PT symmetry coupled microresonator. **a** Optical microscope image of a fabricated PT symmetry coupled dual-microresonators. **b** Typical transmission spectrum of the PT symmetry structure supporting the high-speed signal operation. In this particular case, ${\gamma }_{c}=289$ GHz, $g=120$ GHz forming signal and idler resonances with synthetic linewidths of about 40 GHz. **c** FWM spectrum measured at the output of the bus waveguide by scanning the frequency of the signal light. Colored lines and a black line show the optical spectra when the signal is on resonance and off-resonance, respectively. Colored circles indicate the wavelengths of the signal and the corresponding idler wave. The conversion efficiency is obtained by subtracting the power of the off-resonance signal from the generated idler. The inset shows the zoomed-in spectrum of the generated idler with a black arrow indicating the signal bandwidth. **d** Analytical (lines) and experimental (circles) conversion efficiency plotted as a function of the signal bandwidth at a pump power of 1 mW

Figure 3a shows an optical microscope image of a fabricated coupled AlGaAsOI microresonator with free-spectral ranges of 245 GHz and 490 GHz for the main and auxiliary microresonators, respectively. The fabricated dual coupled microring resonators feature a nominal cross-sectional dimension of 465 $\times$ 290 nm^2^ with an estimated ultrahigh nonlinearity parameter of 720 W^−1^ m^−1^. A microheater is fabricated on top of the main cavity to thermally tune and align the resonances of the two cavities. Figure 3b shows the measured transmission spectrum of the PT symmetry device (solid line) with interleaved high- and low-*Q* resonances, indicating that the signal and idler resonances are deeply over-coupled but the pump resonance is quasi-critically coupled. The measured spectrum agrees well with the simulation result (dotted line, intrinsic *Q* of 8 $\times$ 10^4^ for both the main and auxiliary resonators). The transmission spectrum covering a larger wavelength range is given in Additional file 1: S5.

A series of devices covering a broad range of synthetic signal/idler linewidths has been designed and fabricated. We follow a rough design guideline, i.e., ${\gamma }_{c}=4g=2\uppi B$, to set the signal resonance near the EP with a linewidth corresponding to the expected signal bandwidth of $B$. More precise design guidelines are provided in Additional file 1: S7. Figure 3c shows a frequency-resolved FWM experiment performed by scanning the frequency of a CW signal within one of the broad-linewidth near-EP resonances while another CW pump was tuned into a narrow-linewidth quasi-critical coupled resonance. The profile of the generated idler intensity spectrum is similar to a rectangular shape (zoomed-in inset of Fig. 3c), which is advantageous over the Lorentzian as it minimizes spectral distortion within the resonance. The maximum signal bandwidth for the wavelength conversion is defined as the 3-dB bandwidth of the generated idler intensity spectrum (short for signal bandwidth, illustrated in the inset of Fig. 3c). The conversion efficiency is extracted by subtracting the power (in dBm unit) of the off-resonance signal from the generated idler. The theoretical (solid lines) and experimental (circles) conversion efficiencies of the single cavity (grey) and PT symmetry (purple) devices as a function of signal bandwidth are plotted in Fig. 3d. The theoretical conversion efficiencies are calculated based on two different methods (Additional file 1: S5). The enhancement of conversion efficiency $G$ can be derived by comparing the conversion efficiency of the coupled cavity and the single cavity system designed for the same $B$. Using the models described in Additional file 1: S5, the $G$ can be obtained as,

1 $$G={\left(\frac{{F}_{\text{max}}}{{F}_{B}}\right)}^{2}\approx {\left(\frac{\uppi B}{2{\gamma }_{i1}}\right)}^{2}.$$

For a linewidth-manipulated resonator system (with ${\gamma }_{i}/2\uppi =1$ GHz, equivalently, intrinsic $Q\approx {10}^{5}$) designed for $B=40$ GHz, the bandwidth-efficiency limit of a standard single cavity system can be improved by two orders of magnitude of efficiency enhancement ($G=100$), which is in excellent agreement with the experimental results. The significant enhancement of $\eta$ can also be understood from the fact that $\eta$ is roughly proportional to ${B}^{-4}$ in the single cavity case and ${B}^{-2}$ in the coupled cavity case (Additional file 1: S5).

## Ultra-efficient NOSP system demonstrations

**Fig. 4:**
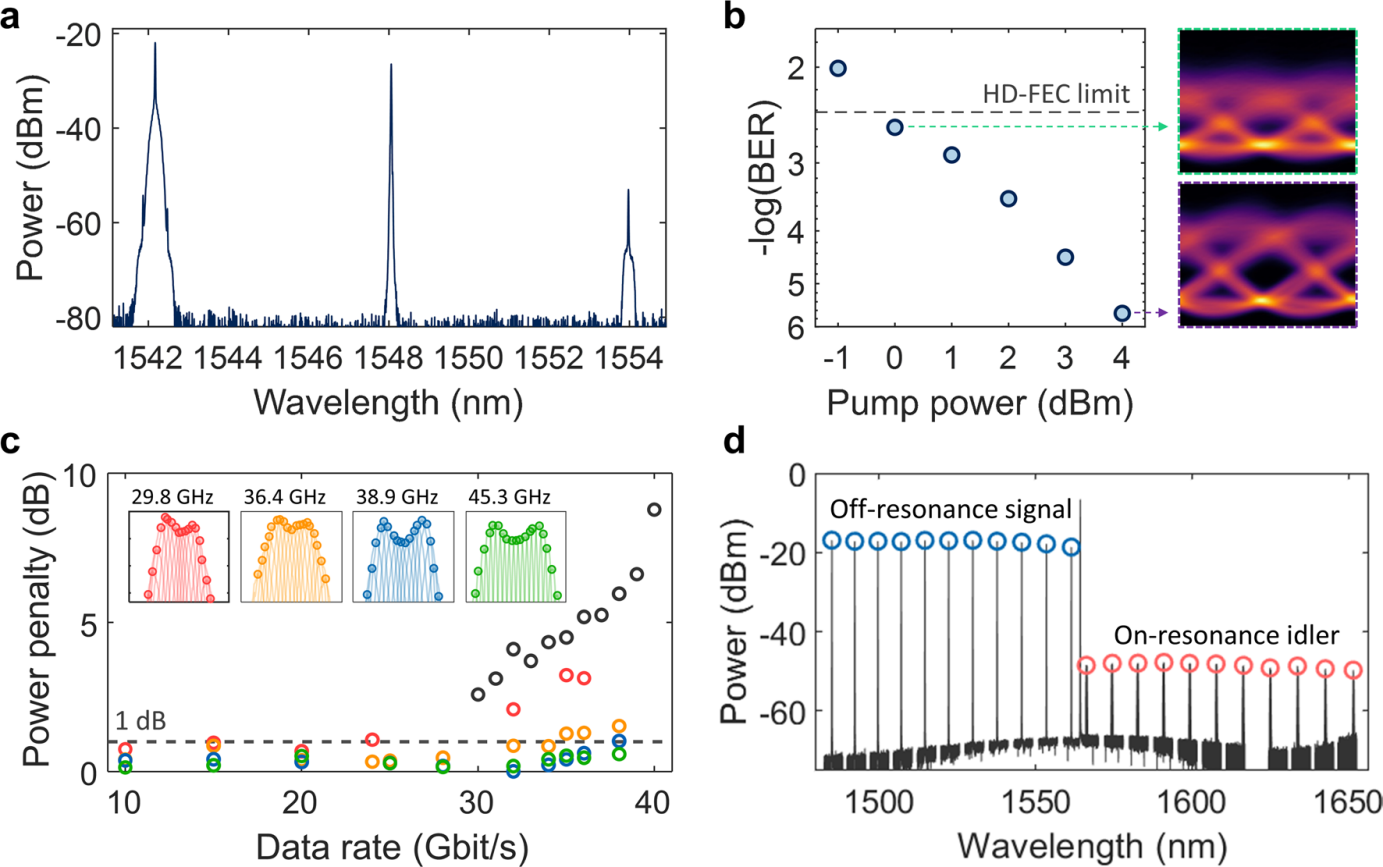
Wavelength conversion of high-speed optical data. **a** Optical spectrum measured at the output of the bus waveguide for the wavelength conversion of 38 Gbit/s on–off keying modulated optical data stream. The optical power at the input of the bus waveguide for the pump and the signal waves are 2.5 mW (4 dBm) and 3.6 mW (5.6 dBm), respectively. **b** Bit-error rate (left) and eye diagram (right) of the converted idler (38 Gbit/s) at different pump power levels with a fixed signal power [given in (**a**)]. **c** Power penalty versus data rates at a hard-decision forward-error coding (HD-FEC) limit for devices with different signal bandwidths of 29.8, 36.4, 38.9, and 45.3 GHz for red, yellow, blue, and green circles, respectively. Signal bandwidths are extracted from the measured conversion efficiency profiles against the frequency detuning of the signal (fixed detuning range of 70 GHz, shown by the inset), following the same procedure as described in Fig. 3c. Black circles indicate the single resonator data points from reference [10], and the dashed line represents a power penalty level of 1 dB. **d** Overlaid FWM spectra with the signal wave tuned into eleven different resonances. The conversion bandwidth measurements show a wavelength conversion operation range of over 170 nm

To validate the device performance in NOSP applications, we experimentally characterize wavelength conversion of high-speed optical data using a nonlinear FWM process. Figure 4a shows the measured FWM optical spectrum at the output of the bus waveguide for the wavelength conversion of a 38 Gbit/s non-return-to-zero on–off keying modulated optical data stream. The bit-error-rates (BERs) of the converted idler data were measured at different pump power levels (Fig. 4b), and a hard-decision forward-error-correction (HD-FEC) limit was achieved with only 1 mW of pump power (see Methods for the system experiment information). We note that the input power of the pump is lower than that of the signal in the wavelength conversion process. It is enabled by the enhanced intracavity intensity of the pump in the main cavity, which is significantly higher than that of the signal owing to the linewidth (enhancement) manipulation. The unique configuration leads to the FWM process in stark contrast to conventional nonlinear optics settings where the light driving the nonlinear phenomenon (i.e., the pump light in our case) has a much higher peak intensity than the other interacting light (i.e., signal light in our case). The effect of pump recycling facilitates the critical coupling of the pump light, offering several notable benefits for both classical and quantum applications. It promotes the use of integrated pump sources by significantly reducing power requirements, while also substantially reducing the unwanted pump component at the output, simplifying the post-filtering (pump-rejection) stage. Figure 4c shows the power penalty (at the HD-FEC limit) of four different devices at different date rates. Negligible power penalties (< 1 dB) at the HD-FEC level are achieved for data rates below the designed bandwidths, indicating much smaller signal distortion (spectral filtering) compared to the results obtained with a single cavity (black circles) [10]. The wavelength conversion bandwidth, determined by the parametric phase-matching bandwidth (related to the group velocity dispersion and the length of the resonator waveguide), is greater than 170 nm (Fig. 4d). Numerical simulations suggest that the conversion range can be further expanded by improving the fabrication accuracy of cavity lengths, introducing wavelength-independent coupling coefficients (see Methods), and engineering the high-order dispersion [49].

In summary, we have demonstrated linewidth manipulation of a photonic resonator system enabled by PT symmetry. Simultaneous broadband and narrowband operation in alternate longitudinal modes via the concept of unbroken and broken PT-symmetry phases addresses the restrictive trade-off relationship between speed and efficiency in cavity-based NOSP systems. With the proposed concept, we experimentally validate a device that is not limited by the traditional performance trade-off constraints, highlighted by its record low pump power of 1 mW driving a high-speed NOSP (data rate of 38 Gbit/s), small footprint (about 0.01 mm^2^), broad wavelength conversion bandwidth (> 170 nm), and ultra-low power-penalty operation (< 1 dB) based on high-nonlinear AlGaAsOI platform. Theoretically, with our device's current intrinsic *Q* factor, a conversion efficiency of 50% (-3 dB) can be achieved with approximately 50 mW of pump power. While this power level is appealing for practical applications, the proof-of-concept device requires further investigation into its nonlinear loss and power handling capabilities. A detailed comparison of state-of-the-art all-optical wavelength conversion system experiment results achieved with different integrated photonic platforms and structures is given in the Additional file 2: Extended Data Fig. S1. We stress that our scheme requires a much lower pump power and shorter device length compared to single-pass waveguide structures and outperforms all the existing cavity-based solutions in terms of power consumption per bit. As the FWM process is transparent to modulation formats and compatible with multichannel operation [14], the demonstrated power consumption per bit of our system could be further lowered by increasing the data throughput (e.g., applying wavelength-division multiplexing and advanced modulation formats) in the experiment. The improvement of the intrinsic *Q* of the main microresonator can greatly enhance the FWM efficiency [see Eq. (1)], where it may facilitate various FWM-enabled NOSP applications such as on-chip amplification/regeneration. The proposed approach paves the way to realizing fully integrated NOSP devices, i.e., a chip-scale NOSP system with an integrated pump source, offering a path to green and high-speed operation of optical communications and computations. In broader scenarios, the potential for non-Hermitian physics to overcome bandwidth-efficiency limits may also have applications in other fields, such as optomechanics [34], quantum optics [50], and atomic physics [51].

## Methods

### Device fabrication details

The fabrication processes for preparing the AlGaAs-on-insulator-based [49] coupled resonator devices are shown in the Additional file 2: Extended Data Fig. S2.

*Sample preparation*. Al_0.21_Ga_0.79_As/InGaP/GaAs/InGaP epitaxial layers are grown on a (100) GaAs wafer through metalorganic vapor-phase epitaxy (Emcore D125). An SiO_2_ cladding layer with a thickness of 3 µm is deposited on top of the epitaxial layers (AlGaAs) using a plasma-enhanced chemical vapor deposition (PECVD) system (SPTS Multiplex PECVD). A carrier wafer (InP) is prepared by depositing 10 nm of SiO_2_ using the PECVD to facilitate the following wafer bonding process.

*Wafer bonding*. The epitaxial wafer is adhesively bonded to the carrier wafer, where both wafers are joined facing the SiO_2_ layers. An adhesion promoter (Dow Corning AP3000) is spin-coated on both bonding interfaces of the wafers, and subsequently, a diluted bisbenzocyclobutene (BCB; Dow Corning Cyclotene 3022–46) using mesitylene (BCB:mesitylene = 1:5) with a thickness of about 100 nm is spin-coated on the carrier wafer side. The wafers are bonded with a wafer bonding system (NILT CPB) at a temperature of 250 °C and a bonding pressure of 6 bar under a vacuum condition for 1 h.

*Substrate removal*. The GaAs substrate and the InGaP/GaAs/InGaP epitaxial layers are removed from the bonded sample leaving the AlGaAs-on-Insulator wafer. The GaAs substrate is rapidly thinned (≈ 3 µm/min.) using a sulfuric acid/hydrogen peroxide (H_2_SO_4_:H_2_O_2_ = 5:4) solution and terminated before the complete removal of the GaAs substrate layer (leaving about 100 µm in thickness). The remaining GaAs substrate is slowly etched and completely removed using a citric acid/hydrogen peroxide solution (C_6_H_8_O_7_:H_2_O_2_ = 4:1). The InGaP/GaAs/InGaP epitaxial layers are removed using hydrogen chloride (HCl) for the InGaP and the C_6_H_8_O_7_:H_2_O_2_ (4:1) solution for the GaAs layer, exposing the AlGaAs thin film.

*Electron-beam lithography*. An electron-beam lithography system (JEOL JBX-9500FS) and a negative electron-beam resist hydrogen silsesquioxane (HSQ; Dow Corning XR-1541) are used to define the device patterns on the AlGaAsOI wafer. A thin (10 nm) layer of SiO_2_ is deposited on AlGaAs film using the PECVD to ensure the adhesion of the resist. The HSQ is spin-coated with a thickness of about 350 nm. In addition, we deposit a layer of aluminum (thickness of 20 nm) using a thermal evaporation system (Kurt J. Lekser Nano 36) to prevent charge-induced pattern distortion during the electron-beam writing process. We use an electron-beam dose of 11,000 µC/cm^2^, a current of 6 nA, and a four-pass exposure to reliably pattern the HSQ. The sample is developed by using a KOH-based developer (MicroChemicals AZ 400 K) diluted using distilled water (AZ 400 K:H_2_O = 1:3) for 190 s.

*Dry etching and top cladding deposition*. The developed pattern is transferred to the AlGaAs thin film using an inductively coupled plasma reactive ion etching system (SPTS ICP). BCl_3_ gas with a flow rate of 20 sccm, chamber pressure of 10 mTorr, platen temperature of 20 °C, coil power of 300 W, and platen power of 50 W. A SiO_2_ layer (top cladding) with a thickness of 1.5 μm is deposited on the device structures using the PECVD.

*Resistive heater definition*. An image-reversal resist AZ 5214e with a thickness of 2.2 µm is spin-coated on the top cladding layer. Heater patterns are aligned to the underlying resonator devices and exposed with ultraviolet (UV) light with a wavelength of 405 nm and a dose of 32 mJ/cm^2^ using a maskless aligner (Heidelberg Instruments MLA150). The sample is baked at 110 °C for 2 min, and flood-exposed with UV light (Süss MicroTec MA6/BA6 aligner) with a wavelength of 365 nm and a dose of 200 mJ/cm^2^. The resist is developed using a 2.38% tetramethyl ammonium hydroxide solution. 10 nm of titanium and 200 nm of platinum layers are deposited on the sample using an electron-beam evaporator (FerroTec Temescal FC2000). The sample is immersed in an n-methyl-2-pyrrolidone solution (Microposit remover 1165) for lift-off.

### System experiment information

The schematic of the wavelength conversion experiment setup is shown in the Additional file 2: Extended Data Fig. S3. The setup is divided into three modules: transmitter, wavelength converter, and receiver.

The transmitter generates an optical data stream with a non-return-to-zero on–off keying (NRZ-OOK) modulation format. A bit pattern generator (SHF BPG44E) generates the NRZ-OOK electrical data signal in a pseudorandom binary sequence with a sequence length of 2^15^–1. The electrical signal is amplified by a radio frequency amplifier (SHF S807) to sufficiently match the ideal driving voltage amplitude of the Mach–Zehnder modulator (Fujitsu FTM7937EZ). The signal light emitted from an external-cavity diode laser (ECDL; Ando AQ4321A) is modulated with the Mach–Zehnder modulator and optically amplified using an Erbium-doped fiber amplifier (EDFA; Amonics AEDFA-C-PA-35-B-FA) with a low noise figure. The optical power of the data-encoded signal light is adjusted with a programmable variable optical attenuator (VOA; HP/Agilent 8156A). The amplified spontaneous emission (ASE) noise from the EDFA is spectrally filtered with a bandpass filter (Koshin Kogaku Tunable Filter) with a bandwidth of 0.8 nm.

The wavelength converter generates an idler light—a phase conjugate replica of the data-encoded signal light—through the degenerate FWM process. Pump light is supplied from an ECDL (Santec TLS550), and lensed fibers are used to couple the lights to and from the coupled resonator device (CR). Polarization controllers (PC) are adjusted to excite the TE_00_ mode of the CR. The resistive heater of the CR is controlled with a power supply (Keithley 2600B). The CR is stabilized in temperature using a closed-loop thermoelectric cooler (Keithley 2510). A bandpass filter rejects the pump and signal light and transmits the idler light (wavelength converted from the signal light). Although omitted in the figure, an optical spectrum analyzer (Yokogawa AQ6370C, Ando AQ6317B) is used to monitor the resonance alignment (through the transmission spectrum of the device) of the main and auxiliary resonator at the signal/idler resonances. When tuning the pump light into the pump resonance, the main resonator will dominantly experience a thermal resonance shift due to the localized pump field in the main resonator. The resonance misalignment between the main and auxiliary resonator caused by the pump is compensated by reducing the applied microheater power on top of the main resonator.

The receiver converts the optical data carried by the idler light into an electrical data signal. A VOA controls the optical power of the idler light (received power) for bit-error-rate (BER) measurements. An EDFA amplifies the idler so that the optical power level is in the optimum detection range of a photodetector (Finisar XPDV2120R) with an electrical bandwidth of 50 GHz. A bandpass filter just before the photodetector filters the ASE noise from the EDFA. A digital storage oscilloscope (Keysight DSOZ634A) with an electrical bandwidth of 63 GHz stores the electrical signal acquired from the photodetector. The stored data signal consisting of four million samples is digitally signal processed offline (brick-wall digital low pass filter and a linear equalization with a tap number of 21) and analyzed for the BER. A corresponding back-to-back experiment (BER measurement of the signal light) was conducted by directly connecting the transmission module to the receiver module.

### Data extraction and normalization of the devices under different coupling conditions

To confirm the PT symmetry feature of our structure, the transmission spectra near the signal resonances of a series of devices operating at different coupling conditions were measured in Fig. 2. Figure 2 shows the evolution of the resonance frequencies and linewidths of the eigenmodes—represented by the normalized real and imaginary parts of the eigenvalues, respectively—as a function of ${\gamma }_{c}/{\gamma }_{c}^{EP}$. Note that the mode splitting is normalized by $Re\left({\omega }_{\pm }-{\omega }_{0}\right)/\left({\gamma }_{c}^{EP}/4\right)$, so that the normalized mode splitting corresponds to ± 1 when ${\gamma }_{c}=0$. Meanwhile, the imaginary part of the eigenvalue is similarly normalized by $Im\left({\omega }_{\pm }\right)/\left(\left({\gamma }_{i1}+{\gamma }_{i2}+{\gamma }_{c}^{EP}\right)/4\right)$, i.e., the normalized $Im\left({\omega }_{\pm }\right)$ corresponds to −1 at the EP (${\gamma }_{c}={\gamma }_{c}^{EP}$). Figure 2b shows the normalized transmission spectra of the devices operating at different coupling conditions, where the blue lines and the red dashed lines denote the experimentally measured data and the theoretical fitting results, respectively. The transmission spectra (i–v) in Fig. 2b correspond to the blue data points in Fig. 2a, in the order of increasing ${\gamma }_{c}/{\gamma }_{c}^{EP}$. The blue data points in Fig. 2a are the normalized mode splitting or imaginary parts of the eigenvalues obtained from curve-fitting each measured transmission spectrum to the analytical model (Additional file 1: S1, S2). That is, the transmission spectrum is fitted using TMM to extract the roundtrip field attenuation factor of cavities, field coupling coefficients between the resonators as well as between the auxiliary and main cavity. Intrinsic decay rate of the two resonators ${\gamma }_{i1, i2}$, energy coupling rate $g$ and coupling rate between auxiliary cavity and the bus waveguide ${\gamma }_{c}$ are derived from TMM according to Additional file 1: Table S1. TCMT is then applied to extract data points shown in Fig. 2a using Additional file 1: Eq. (S6).

### Phase-matching bandwidth

The synthetic linewidths—governed by the resonant effect—designed for the signal and idler waves determine the signal bandwidth (or data rates) of the optical signal that can be processed by our devices. Phase-matching bandwidth (determined by the dispersion) is another important parameter in NOSP applications that describes the wavelength conversion ranges of the devices, i.e., the maximum available wavelength (or frequency) distance between the input signal and generated idler. The phase-matching conversion bandwidth is commonly defined as the 3-dB bandwidth of the envelope of the FWM conversion efficiency $\eta$ calculated according to Additional file 1: S4 (Eq. S26) when many resonances are considered. Normalized FWM conversion efficiency is shown in Additional file 2: Extended Data Fig. S4b. The experimental data (blue circles, obtained from Fig. 4d) and a simulation curve (red line) are plotted together with respect to the signal wavelength. The following parameters are used for simulations. The propagation loss ${\alpha }_{1}$ is 4.5 dB/cm, the resonator length ${L}_{1}$ is 302 µm, and the group velocity dispersion (GVD, related to phase mismatch factor $\Delta k$) is numerically (Lumerical MODE solutions) obtained for a fundamental TE mode of an AlGaAsOI waveguide with a width of 465 nm and a thickness of 290 nm. The GVD used in the simulation is plotted in Additional file 2: Extended Data Fig. S4a. The narrower bandwidth in the experiment result (~ 173.6 nm) compared to the simulation (~ 291.7 nm) in Additional file 2: Extended Data Fig. S4b may be attributed to the (1) wavelength-dependent coupling coefficients and (2) slight resonance misalignment between the main and auxiliary resonator.

### Comparison with the state-of-the-art wavelength conversion experiments

The comparison of state-of-the-art all-optical wavelength conversion system experiment results achieved with different integrated photonic platforms and structures [9, 10, 12, 16, 19, 36, 49, 52–65] is shown in the Additional file 2: Extended Data Fig. S1. Our PT symmetry design outperforms all the existing cavity-based solutions in terms of power consumption per bit, i.e., power divided by data rate. The power consumption per bit of our system, i.e., the pump power divided by the data rate, is as low as 26 fJ/bit, which is comparable to the best result achieved for the waveguide devices, and could be further lowered by increasing the data throughput. We stress that our scheme requires a much lower level of absolute pump power and shorter device length compared to single-pass waveguide structures.

According to the toy model described in Additional file 1: S5 (Eqs. S20, S21), the relationship between the bandwidth $B$ and the conversion efficiency $\eta$ can be expressed as:

$$\begin{array}{c}{\eta }_{single}=\frac{16{\left|\gamma P{L}_{eff}\right|}^{2}{FSR}_{1}^{4}}{{\left(\pi B\right)}^{4}}\\ {\eta }_{dual}=\frac{4{\left|\gamma P{L}_{eff}\right|}^{2}{FSR}_{1}^{4}}{{\left({\gamma }_{i1}\pi B\right)}^{2}}\end{array}$$

where ${\eta }_{single}$ is the conversion efficiency of the single resonator system under the bandwidth $B$, and ${\eta }_{dual}$ is the maximum conversion efficiency of our dual resonator system under the same bandwidth.

For the single resonator system:

$${B}^{4}\cdot {\eta }_{single}={B}^{4}{\left|\gamma P{L}_{eff}\right|}^{2}{F}_{p,s,i\_single}^{4}\approx {\left|\gamma P{L}_{eff}\right|}^{2}{\left(4{FSR}_{1}\right)}^{4}$$

For our system:

$${B}^{2}\cdot {\eta }_{dual}={B}^{2}{\left|\gamma P{L}_{eff}\right|}^{2}{F}_{pmax\_dual}^{2}{F}_{s,i\_dual}^{2}\approx {\left|\gamma P{L}_{eff}\right|}^{2}{\left(4{FSR}_{1}\right)}^{2}{\left(\frac{{FSR}_{1}}{{\gamma }_{i1}}\right)}^{2}$$

Since the maximum processing data rate $D$ can be roughly approximated as bandwidth $B$, i.e., $D\approx B$, we can get:

$$\begin{array}{c}{D}^{4}\cdot {\eta }_{single}\approx {constant}_{1}\cdot {P}^{2}\\ {D}^{2}\cdot {\eta }_{dual}\approx {constant}_{2}\cdot {P}^{2}\end{array}$$

As a rough estimation, the receiver sensitivity and processed data rate can be viewed as linearly related. Therefore, the required conversion efficiency of the received signal to achieve identical BER is linearly related to its data rate, i.e., $\eta \propto D$, assuming the same input signal power. Thereby, the relationship between the data rate and pump power of the single resonator system can be obtained as:

$${D}^{4}\cdot D\approx {constant}_{3}\cdot {P}^{2}$$

In the logarithmic axis, the relationship between the data rate and pump power of the single resonator system is given by:

$$log\left(D\right)\approx \frac{2}{5}log\left(P\right)+\frac{1}{5}log\left({constant}_{3}\right)$$

For our system, the relationship between the data rate and pump power can be expressed as:

$$\begin{array}{c}{D}^{2}\cdot D\approx {constant}_{4}\cdot {P}^{2}\\ log\left(D\right)\approx \frac{2}{3}log\left(P\right)+\frac{1}{3}log\left({constant}_{4}\right)\end{array}$$

Therefore, the data rate/power slope of our system is 2/3, which is higher than 2/5 of the single cavity, indicating the superiority of our system in terms of power consumption per bit. The slopes of the solid and dashed red lines in Additional file 2: Extended Data Fig. S1 correspond to 2/3 and 2/5, respectively. Our PT-symmetry system breaks the data rate-power limit of the single ring, dramatically reducing the required pump power for high-speed wavelength conversion in resonator systems. For instance, the required pump power of wavelength conversion at a data rate approaching 40 Gbit/s has been reduced from 32 mW (single resonator based on AlGaAsOI [10]) to 1 mW (our coupled resonator based on AlGaAsOI).

### Supplementary Information

**Additional file 1:** **Section S1**. Temporal coupled mode theory (TCMT).** Fig. S1**. Schematic diagram of a single micro-ring resonator coupled to a bus waveguide. **Fig. S2**. Notations of PT-symmetric structure under TCMT. **Section S2**. Transfer matrix method (TMM).** Fig. S3**. Notations of PT-symmetric structure under TMM. **Tab. S1**. The relationships between the parameters of the TMM and TCMT. **Section S3**. Comparison between TCMT and TMM. **Fig. S4**. Evolution of normalized transmission spectrum with varying ${\gamma }_{c}/{\gamma }_{c}^{{\text{EP}}}$. **Fig. S5**. The comparison results of the intensity enhancement spectrum between the TMM and TCMT in the main resonator at the signal resonance. **Fig. S6**. Transmission of the PT symmetry structure at the pump wavelength as a function of *k*_2_ and *k*_3_ using TMM. **Fig. S7**. Comparison results between the intensity enhancement spectra of the pump wave in the main resonator using TMM (solid blue lines) and the spectra of a single cavity under critical coupling using $F_{single{-}critical}$ (${\omega }$) (dashed red lines) at various design signal bandwidths. **Fig. S8**. Illustration of the critical coupling condition of the pump light from the PT-symmetry breaking point of view. **Fig. S9**. The comparison results of the intensity enhancement spectrum between the TCMT and TMM in the main resonator at the pump resonance. **Fig. S10**. Comparison results of intensity enhancement spectrum between the TMM and TCMT of the pump wave in the main resonator when different signal bandwidths are realized. **Section S4**. Analytical model of FWM conversion efficiency. **Section S5**. Full-map coupled nonlinear Schrödinger equations. **Fig. S11**. Comparison of full-map model and TMM. **Fig. S12**. Transmission measured from dual-coupled cavity system. **Fig. S13**. Comparison between conversion efficiency derived by TMM and full-map model. **Section S6**. Synthetic linewidth of dual cavity. **Fig. S14**. Evolution of the system synthetic linewidth with varying ${\gamma }_{c}/{\gamma }_{c}^{{\text{EP}}}$. **Section S7**. Design guideline. **Fig. S15**. Performance comparison of a coupled resonator and an all-pass single microresonator system. **Fig. S16**. Design guideline of the PT symmetry system. **Section S8**. Intracavity field distribution. **Fig. S17**. Simulated intracavity field distribution normalized by the input field amplitude. ** Section S9**. PT-symmetry features by varying the intracavity coupling rate. **Fig. S18**. Evolution of the transmission spectrum and the eigenfrequencies of signal/idler resonances as a function of coupling rate *g*.

**Additional file 2: **Extended Data **Fig. S1.** Performance comparison of reported all-optical wavelength conversion systems realized on integrated photonic platform. Rectangular, circular, star-shaped markers represent the type of the reported devices: waveguides, resonators, and this work, respectively. The color shading of the markers indicates the device lengths.  Extended Data **Fig. S2.** Fabrication process of coupled resonator devices.  Extended Data **Fig. S3.** Schematic of the wavelength conversion experimental setup. ECDL external cavity diode laser, PC polarization controller, MZM Mach-Zehnder modulator, RFA radio frequency power amplifier, BPG bit pattern generator, EDFA Erbium-doped fiber amplifier, VOA variable optical attenuator, BPF bandpass filter, CR coupled resonator device, PD photodiode, and DSO digital storage oscilloscope.  Extended Data **Fig. S4.** FWM wavelength conversion (phase-matching) bandwidth. a Simulated group velocity dispersion (GVD) of the AlGaAsOI waveguide with a width of 465 nm and a thickness of 290 nm. b Red line is the simulated normalized conversion efficiency according to the GVD information in a, and blue circles are the conversion efficiency data points extracted from Fig. 4d.

## Data Availability

The datasets generated during and/or analysed in this study are available from the corresponding author upon reasonable request.
