# Supplementary material for: Parity-time symmetry enabled ultra-efficient nonlinear optical signal processing
Source: eLight. 2024 Apr 4;4(1):6. doi: 10.1186/s43593-024-00062-w (PMC10995095; doi:10.1186/s43593-024-00062-w)
Supplement: Supplementary file 1 — Additional file 1: Section S1. Temporal coupled mode theory (TCMT). Fig. S1. Schematic diagram of a single micro-ring resonator coupled to a bus waveguide. Fig. S2. Notations of PT-symmetric structure under TCMT. Section S2. Transfer matrix method (TMM). Fig. S3. Notations of PT-symmetric structure under TMM. Tab. S1. The relationships between the parameters of the TMM and TCMT. Section S3. Comparison between TCMT and TMM. Fig. S4. Evolution of normalized transmission spectrum with varying ${\gamma }_{c}/{\gamma }_{c}^{{\text{EP}}}$γc/γcEP. Fig. S5. The comparison results of the intensity enhancement spectrum between the TMM and TCMT in the main resonator at the signal resonance. Fig. S6. Transmission of the PT symmetry structure at the pump wavelength as a function of k2 and k3 using TMM. Fig. S7. Comparison results between the intensity enhancement spectra of the pump wave in the main resonator using TMM (solid blue lines) and the spectra of a single cavity under critical coupling using $F_{single{-}critical}$Fsingle-critical (${\omega }$ω) (dashed red lines) at various design signal bandwidths. Fig. S8. Illustration of the critical coupling condition of the pump light from the PT-symmetry breaking point of view. Fig. S9. The comparison results o [file 43593_2024_62_MOESM1_ESM.docx]

**Additional file for**  **Parity-time symmetry enabled ultra-efficient nonlinear optical signal processing**

Chanju Kim,^1,2†^ Xinda Lu,^1,2†^ Deming Kong,^2^ Nuo Chen,^1^ Yuntian Chen,^1,3^ Leif Katsuo Oxenløwe,^2^ Kresten Yvind,^2^ Xinliang Zhang, ^1,3,4^ Lan Yang^5^, Minhao Pu^2^* and Jing Xu^1,3,4^*

^1^ School of Optical and Electronic Information, Huazhong University of Science and Technology, Luoyu Road 1037#, Wuhan 430074, China

^2^ DTU Electro, Department of Electrical and Photonics Engineering, Technical University of Denmark, Øresteds Plads 343, 2800 Kongens Lyngby, Denmark

^3^ Wuhan National Laboratory for Optoelectronics, Huazhong University of Science and Technology, Luoyu Road 1037#, Wuhan 430074, China

^4^ Optics Valley Laboratory, Hubei 430074, China

^5^ Department of Electrical and Systems Engineering, Washington University, St. Louis, MO 63130, USA.

Contents

S1. Temporal coupled mode theory (TCMT) 1

S2. Transfer matrix method (TMM) 4

S3. Comparison between TCMT and TMM 6

S4. Analytical model of FWM conversion efficiency 13

S5. Full-map coupled nonlinear Schrödinger equations 17

S6. Synthetic linewidth of dual cavity 20

S7. Design guideline 21

S8. Intracavity field distribution 23

S9. PT-symmetry features by varying the intracavity coupling rate 25

References: 27

# S1. Temporal coupled mode theory (TCMT)

We introduce a theoretical framework for describing the parity-time (PT) symmetric structure shown in **Fig. 1b** using TCMT^1^. First, we start with the description of a single micro-ring resonator coupled to a bus waveguide (**Fig. S1**). We define the intracavity mode field of the resonator as $a_{1}$with a resonance frequency $\omega_{1}$, the coupling rate between the resonator and the waveguide as $\gamma_{\text{c1}}$, the intrinsic decay rate of the resonator as $\gamma_{\text{i1}}$, the free spectral range (FSR) of the resonator as $FSR_{1}$, the input field as $a_{in}$, and the output field as $a_{out}$.


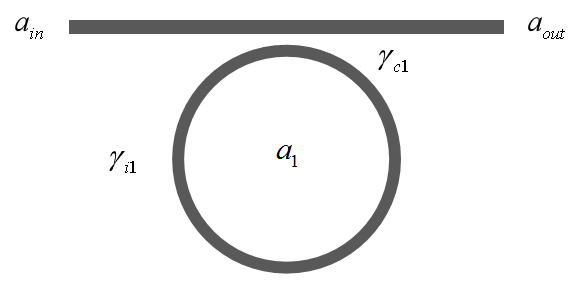


**Fig. S1 |** **Schematic diagram of a single micro-ring resonator coupled to a bus waveguide.**

The coupled mode equations can be written as:

$\begin{matrix} \frac{da_{1}}{dt}=-i\omega_{1}a_{1}-\frac{\gamma_{i1}+\gamma_{c1}}{2}a_{1}-\sqrt{\gamma_{c1}}a_{in} \\ a_{\text{out}}=a_{in}+\sqrt{\gamma_{c1}}a_{1} \end{matrix}$. (S1)

The steady-state transmission spectrum, as well as intensity enhancement, are given by:

$\begin{matrix} T=\left|\frac{A_{\text{out}}}{A_{in}}\right|^{2}=\left|1+\frac{2\gamma_{c1}}{2i\Delta_{1}-\left(\gamma_{i1}+\gamma_{c1}\right)}\right|^{2} \\ F=\left|\frac{A_{1}}{A_{in}}\right|^{2}=\left|\frac{2\sqrt{\gamma_{c1}}}{2i\Delta_{1}-\left(\gamma_{i1}+\gamma_{c1}\right)}\right|^{2}FSR_{1} \end{matrix}$ (S2)

where $\Delta_{1}$ is the frequency detuning of the excitation frequency $\omega$ from the resonant frequency $\omega_{1}$, which is expressed as $\Delta_{1}=\omega-\omega_{1}$.


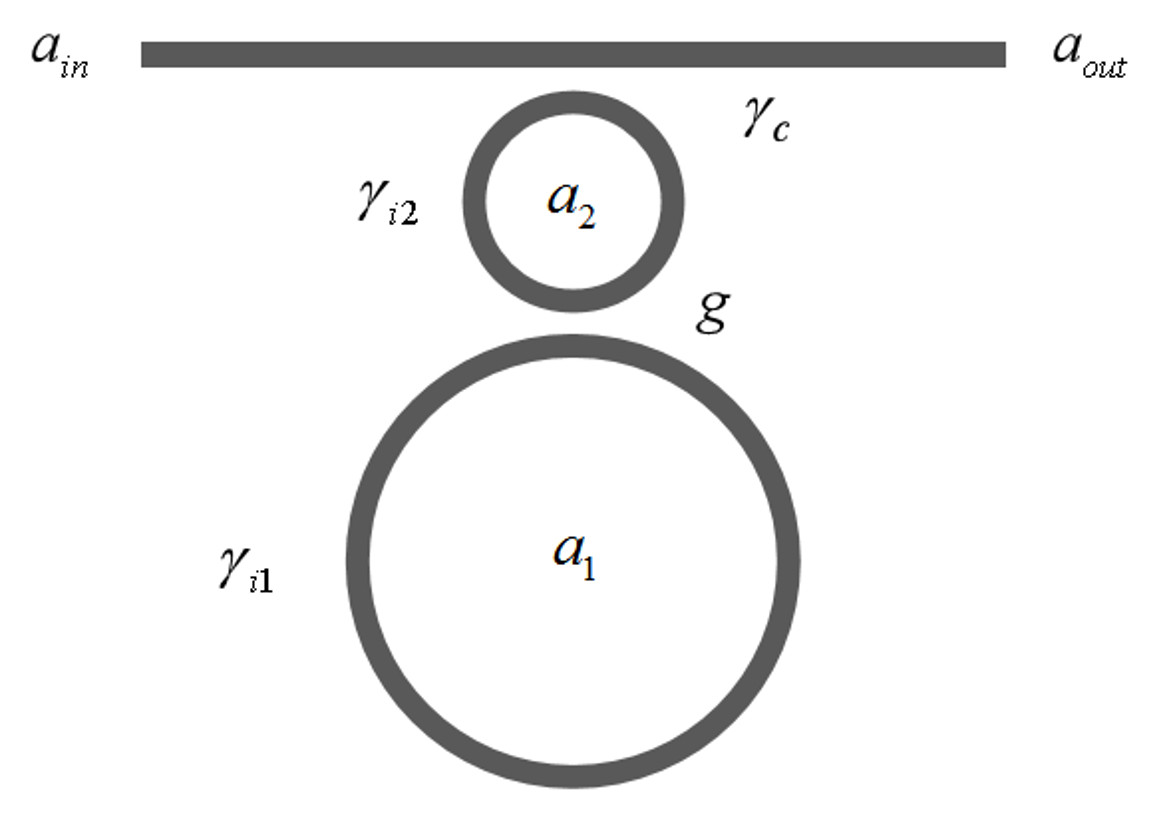


**Fig. S2 | Notations of PT-symmetric structure under TCMT.**

Next, we discuss the TCMT for our structure (**Fig. S2**). The coupled mode equations can be written as^1^:

$\begin{matrix} \frac{da_{1}}{dt}=-i\omega_{1}a_{1}-\frac{\gamma_{i1}}{2}a_{1}-iga_{2} \\ \frac{da_{2}}{dt}=-i\omega_{2}a_{2}-\frac{\gamma_{i2}+\gamma_{c}}{2}a_{2}-iga_{1}-\sqrt{\gamma_{c}}a_{in} \\ a_{\text{out}}=a_{in}+\sqrt{\gamma_{c}}a_{2} \end{matrix}$, (S3)

where $a_{\text{k=1,2}}$ are the intracavity mode fields of the resonators, and $\omega_{\text{k=1,2}}$ are the resonance frequencies of the resonators. The subscript index $k$ represents the main ($k=1$) and auxiliary ($k=2$) resonators; $a_{in}$ and $a_{out}$ are the input and output fields of the bus waveguide, respectively. Other parameters are defined in the main text. According to Eq. (S3), the modal field vector $A=\left(\begin{matrix} a_{1} & a_{2} \end{matrix}\right)^{T}$ obeys $idA/dt=HA-i\sqrt{\gamma_{c}}\left(\begin{matrix} 0 & a_{in} \end{matrix}\right)^{T}$, where the non-Hermitian Hamiltonian $H$ is expressed in the following form,

$H=\left[\begin{matrix} \omega_{1}-i\gamma_{i1}/2 & g \\ g & \omega_{2}-i\left(\gamma_{i2}+\gamma_{c}\right)/2 \end{matrix}\right]$. (S4)

The coupled supermodes are characterized by the complex eigenfrequencies expressed as

$\omega_{\pm}=\left[\frac{1}{2}\left(\omega_{1}+\omega_{2}\right)-\frac{i}{4}\left(\gamma_{i1}+\gamma_{i2}+\gamma_{c}\right)\right]\pm\frac{1}{4}\sqrt{16g^{2}-\left[2i\left(\omega_{1}-\omega_{2}\right)-\left(\gamma_{i2}+\gamma_{c}-\gamma_{i1}\right)\right]^{2}}$. (S5)

When the resonance of the two resonators aligns with each other, i.e., $\omega_{1}=\omega_{2}=\omega_{0}$, the eigenfrequencies can be rewritten as

$\omega_{\pm}=\left[\omega_{0}-\frac{i}{4}\left(\gamma_{i1}+\gamma_{i2}+\gamma_{c}\right)\right]\pm\frac{1}{4}\sqrt{16g^{2}-\left(\gamma_{i2}+\gamma_{c}-\gamma_{i1}\right)^{2}}$. (S6)

The exceptional point (EP) of the system occurs at $\gamma_{c}+\gamma_{1}+\gamma_{2}=4g$. Since$\gamma_{c}>>\gamma_{1}\approx\gamma_{2}$, the EP occurs approximately at$\gamma_{c}=\gamma_{c}^{EP}\approx4g$.

Eq. (S3) can be transformed into the following form by substituting $a_{\text{k}}=A_{k}e^{-i\omega t}$ and $\frac{da_{\text{k}}}{dt}=-i\omega A_{k}e^{-i\omega t}+\frac{dA_{\text{k}}}{dt}e^{-i\omega t}$:

$\begin{matrix} \frac{dA_{1}}{dt}=i\Delta_{1}A_{1}-\frac{\gamma_{i1}}{2}A_{1}-igA_{2} \\ \frac{dA_{2}}{dt}=i\Delta_{2}A_{2}-\frac{\gamma_{i2}+\gamma_{c}}{2}a_{2}-igA_{1}-\sqrt{\gamma_{c}}A_{in} \\ A_{\text{out}}=A_{in}+\sqrt{\gamma_{c}}A_{2} \end{matrix}$, (S7)

where $\Delta_{k}=\omega-\omega_{k}$ is the frequency detuning between the resonance frequencies $\omega_{k}$ and the frequency of the input light $\omega$. The transmission spectrum ($T$), intensity enhancements in the auxiliary cavity ($F_{aux}$) and main cavity ($F_{main}$) are derived from Eq. (S7) and written as:

$\begin{matrix} T=\left|\frac{A_{\text{out}}}{A_{in}}\right|^{2}=\left|1+\frac{2\gamma_{c}\left(2i\Delta_{1}-\gamma_{i1}\right)}{4g^{2}+\left(2i\Delta_{1}-\gamma_{i1}\right)\left(2i\Delta_{2}-\left(\gamma_{i2}+\gamma_{c}\right)\right)}\right|^{2} \\ F_{aux}=\left|\frac{A_{2}}{A_{in}}\right|^{2}=\left|\frac{2\sqrt{\gamma_{c}}\left(2i\Delta_{1}-\gamma_{i1}\right)}{4g^{2}+\left(2i\Delta_{1}-\gamma_{i1}\right)\left(2i\Delta_{2}-\left(\gamma_{i2}+\gamma_{c}\right)\right)}\right|^{2}FSR_{2} \\ F_{main}=\left|\frac{A_{1}}{A_{in}}\right|^{2}=\left|\frac{4ig\sqrt{\gamma_{c}}}{4g^{2}+\left(2i\Delta_{1}-\gamma_{i1}\right)\left(2i\Delta_{2}-\left(\gamma_{i2}+\gamma_{c}\right)\right)}\right|^{2}FSR_{1} \end{matrix}$ (S8)

where $FSR_{\text{k=1,2}}$ is the FSR of the resonators.

# S2. Transfer matrix method (TMM)


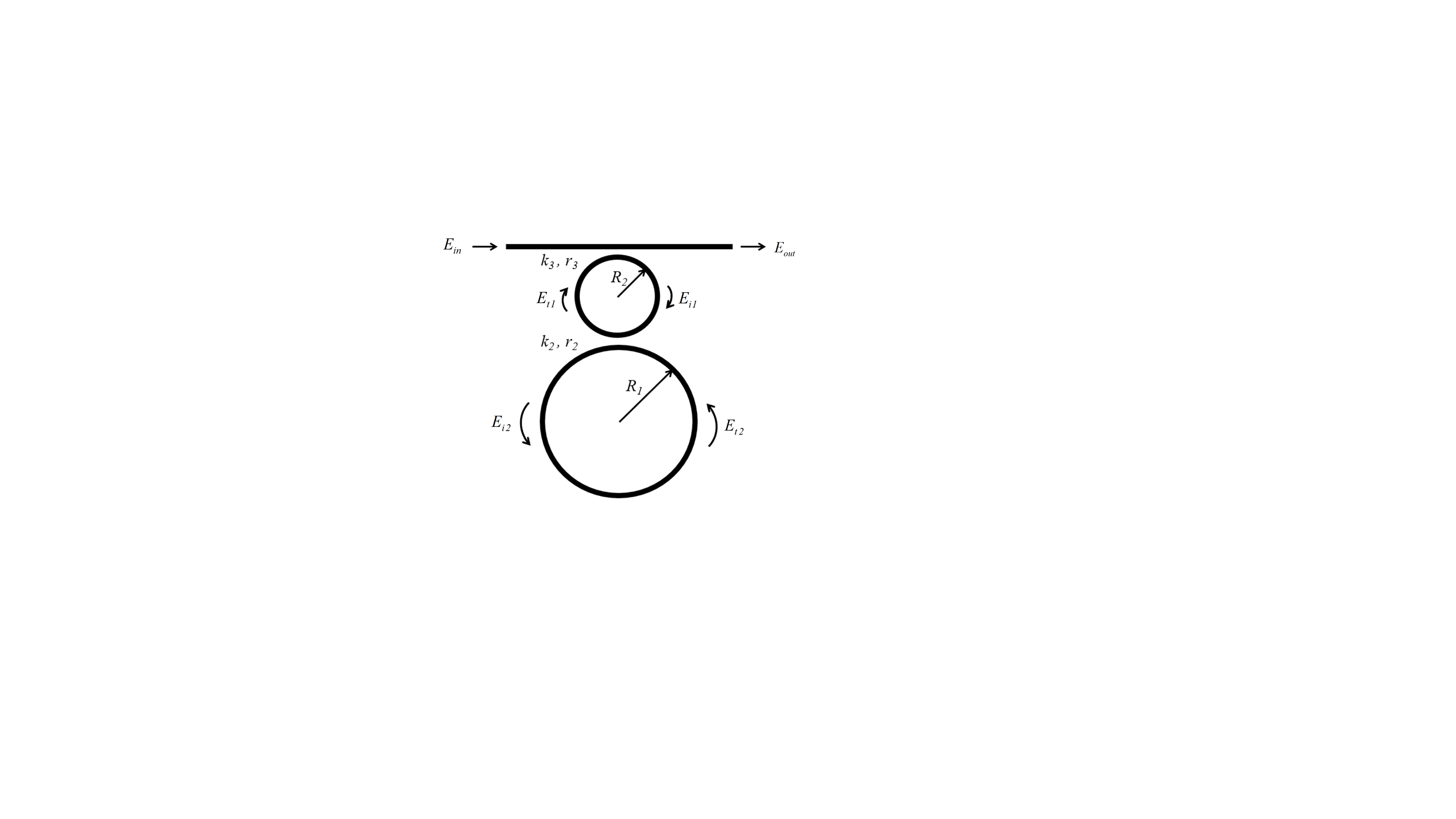


**Fig. S3 | Notations of PT-symmetric structure under TMM.**

The coupled dual-ring structure can be rigorously analyzed using the transfer matrix method (TMM)^2^**. Fig. S3** shows the notations used in the TMM, where $k_{2}$ is the field coupling coefficient between the resonators, $k_{3}$ is the field coupling coefficient between the auxiliary resonator and the waveguide, $r_{j=2,3}$is the transmission coefficient, $E_{i1} ,E_{t1}$ are the fields in the auxiliary resonator, $E_{i2} ,E_{t2}$ are the fields in the main resonator, $E_{in}$ is the input field, and $E_{out}$ is the output field at the bus waveguide.

The transmission spectrum of the structure and intensity enhancement of the resonators can be expressed as

, (S9)

where $\sigma_{k=1,2}$ is the roundtrip field attenuation factor of the resonators, is the phase shift of input light traveling per roundtrip in the resonators and . The conversion relationship between parameters of the TMM and TCMT are listed in **Tab. S1**^2^.

| TMM | | TCMT | | Conversion relationship |
| --- | --- | --- | --- | --- |
| Field coupling coef. between the resonators | $k_{2}$ | Energy coupling rate between the resonators | $g$ | $g=k_{2}\cdot\frac{c}{n_{0}\sqrt{L_{1}L_{2}}}$ |
| Field coupling coef. between the aux. resonator and the waveguide | $k_{3}$ | Coupling rate between the aux. resonator and the waveguide | $\gamma_{c}$ | $\gamma_{c}=-\ln\left(r_{3}\right)\cdot\frac{2c}{n_{0}L_{2}}$ |
| Field transmission coef. of coupling region | $r_{j=2,3}$ | - | - | $r_{j}^{2}+k_{j}^{2}=1$ |
| Roundtrip field attenuation factor of the resonator $k$ | $\sigma_{k=1,2}$ | Intrinsic decay rate of the resonator $k$ | $\gamma_{ik}$ | $\gamma_{ik}=-\ln\left(\sigma_{k}\right)\cdot\frac{2c}{n_{0}L_{k}}$ |

**Tab. S1: The relationships between the parameters of the TMM and TCMT.** $L_{k=1,2}$**is the length of the resonator, $k$, $n_{0}$ is the effective mode index of the resonators, and $c$ is the speed of light.**

# S3. Comparison between TCMT and TMM

**Signal and idler resonances**

First, we analyze the operation of the signal and idler wave using TCMT. **Figure S4a** shows the normalized transmission spectra obtained from Eq. (S8) as a function of $\gamma_{c}/\gamma_{c}^{EP}$when the two cavities are aligned, i.e., $\Delta_{1}=\Delta_{2}$. Here, the transmission spectrum is linearly normalized so that the maxima and minima correspond to 1 and 0, respectively. **Figure S4b** shows simulated 2D transmission plot at five different locations, i.e., **i-v** marked in **Fig. S4a**, where the experimental results are shown in **Fig. 2b**. The white and purple lines shown in **Fig. S4a** correspond to $\mathrm{Re}\left(\omega_{\pm}\right)$ and the half-transmittance of the normalized transmission spectrum, respectively, and correspond to the gray and purple lines in **Fig. 2a**, respectively. The shallowing of the profile at the center of the split resonance – notch depth of the resonance – becomes smaller than 3 dB in the region approaching the EP (bifurcation point of the white line, $\gamma_{c}/\gamma_{c}^{EP}=1$), forming a broad flat-bottom resonance profile suitable for supporting the broadband signal/idler waves (blue arrows, **Fig. S4b‑iii**). A low-loss supermode is formed in the PT-symmetry broken regime (after the EP, $\gamma_{c}/\gamma_{c}^{EP}>1$) with a narrower linewidth (**Fig. S4b-v**) and a localized field distribution in the main cavity.


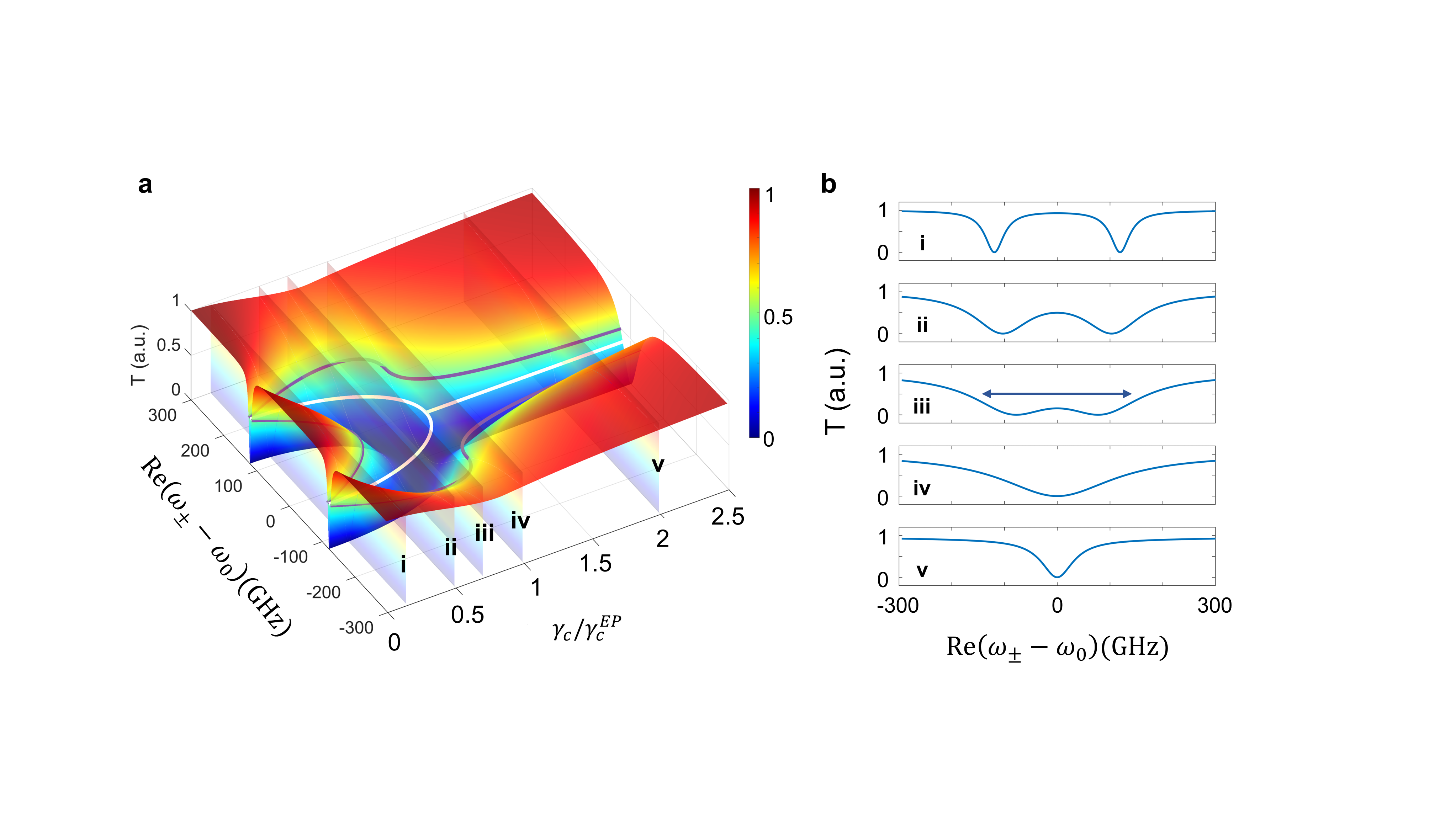


**Fig. S4 | Evolution of normalized transmission spectrum with varying** $\gamma_{c}/\gamma_{c}^{EP}$**. a**, Surface plot of the transmission spectra obtained from Eq. (S8) as a function of $\gamma_{c}/\gamma_{c}^{EP}$. **b**, Transmission spectra at positions **i, ii, iii, iv**, and **v** (from top to bottom). The white lines and the purple lines correspond to $\mathrm{Re}\left(\omega_{\pm}-\omega_{0}\right)$ and half-transmittance of the normalized transmission spectrum, respectively.

Next, TMM is applied to predict the signal/idler resonances using Eq. (S9). The $k_{2}$ and $k_{3}$ used for the TMM analysis are derived from the corresponding $g$ and $\gamma_{c}$ according to the **Tab. S1**. **Figure S5** compares the intensity enhancement of the signal resonance obtained with two methods, where the blue and red lines represent the intensity enhancements calculated by TMM and TCMT, respectively. The consistency between the results verifies the validity of the two methods.


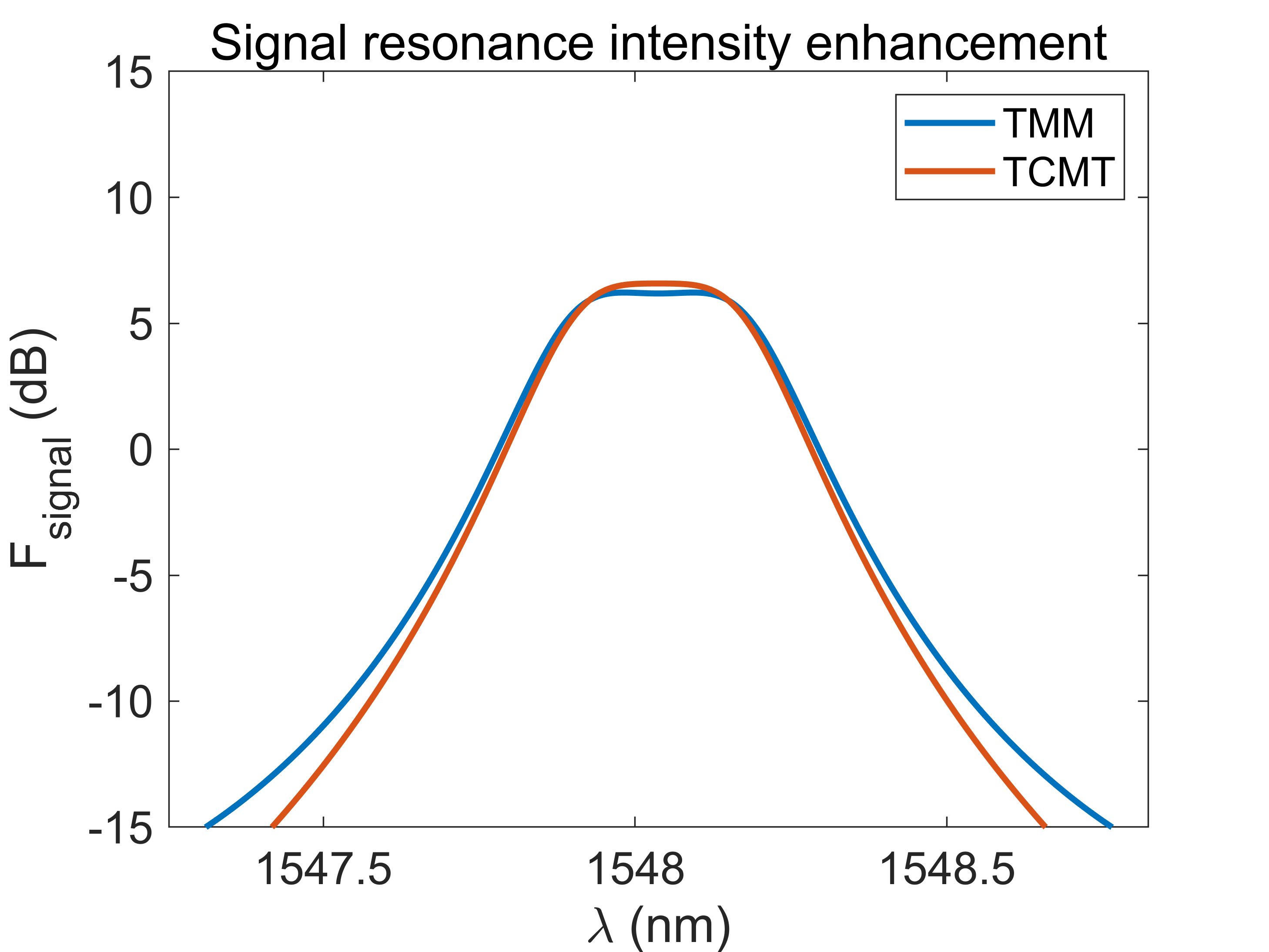


**Fig. S5 | The comparison results of the intensity enhancement spectrum between the TMM and TCMT in the main resonator at the signal resonance.** Parameters for the TMM coupling coefficient are$k_{2}$=0.3984, and $k_{3}$=0.7387. Parameters for the TCMT coupling decay are$g$= 138 GHz, $\gamma_{c}$= 387 GHz. The intrinsic decay rate of the main and auxiliary resonators used in the two different methods are the same, with a value of 8.75 GHz.

**Pump resonance**

First, we discuss the operation condition for the pump frequency using TMM. As discussed in the main text, our device's optimal performance is achieved when the pump light operates at the critical coupling condition, which is equivalent to setting the transmission spectrum $T=0$ in Eq. (S9) at the pump resonance wavelength. Thus, the required condition for the critical coupling of the pump light can be obtained as:

$r_{3}=\frac{\left(\sigma_{1}-r_{2}\right)\sigma_{2}}{1-r_{2}\sigma_{1}}$. (S10)

When the pump light achieves the critical coupling condition, extremely high intensity enhancement can be achieved in the main cavity. **Figure S6** shows the transmission at the pump frequency as a function of $k_{2}$ and $k_{3}$. The red line in **Fig. S6** represents the critical coupling condition given by Eq. (S10). It is shown that the quasi-critical coupling condition, i.e. $T\to0$, can be achieved in a wide combination of $k_{2}$ and $k_{3}$(deep blue region).


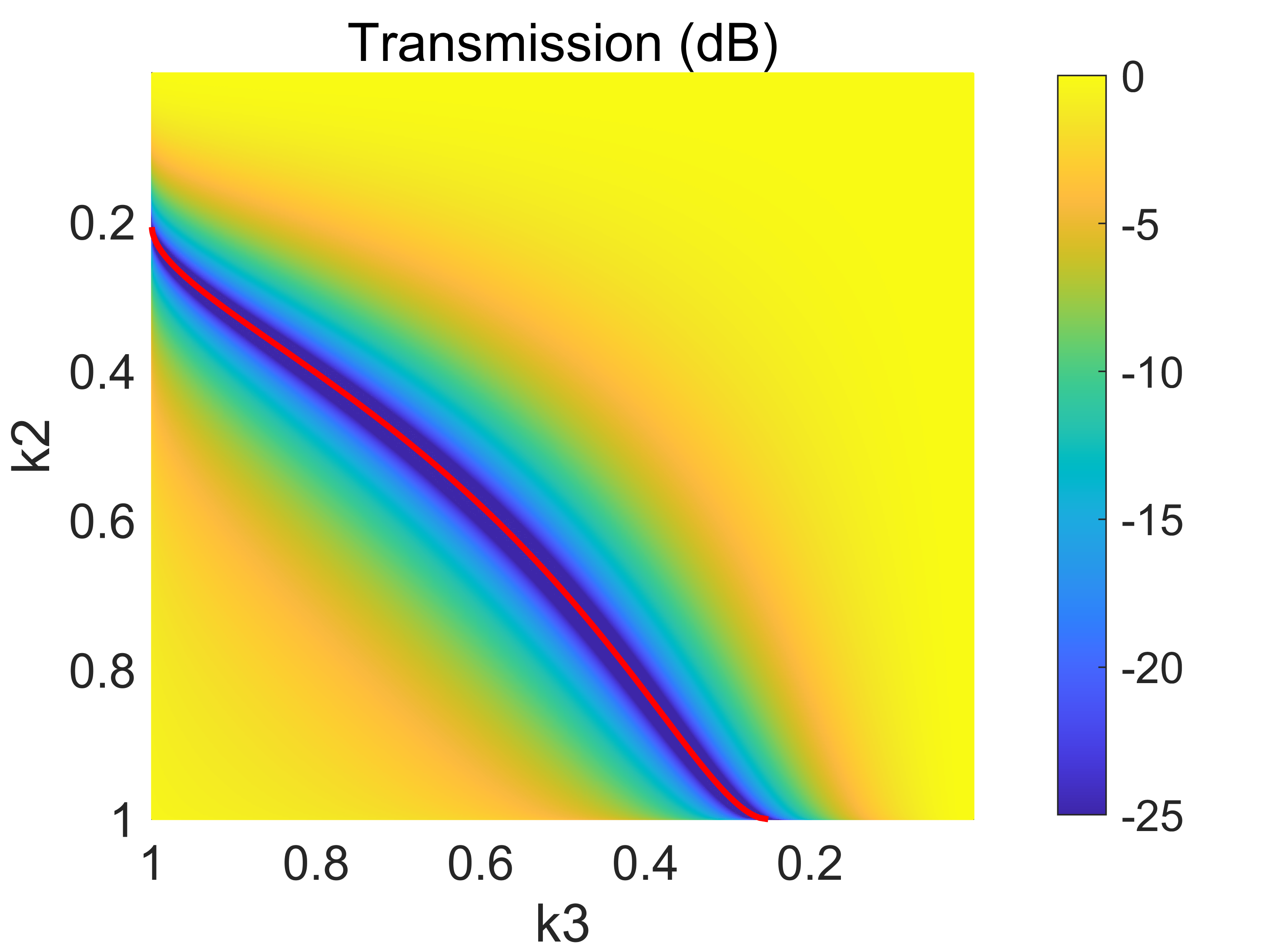


**Fig. S6 | Transmission of the PT symmetry structure at the pump wavelength as a function of** $k_{2}$ and $k_{3}$**using TMM.** Red line is calculated according to Eq. (S10).

Since different signal bandwidth is achieved at different combinations of $k_{2}$ and $k_{3}$, we further show that the quasi-critical coupling can be achieved for the pump wave over a wide range of designed signal bandwidths. Specifically, we present the intensity enhancement spectra of the pump resonance obtained from the TMM at different signal bandwidths (**Figure S7,** solid blue lines). We compare these to the intensity enhancement spectra of the single resonator system (with the same cavity length as the main resonator) under the critical coupling (dashed red lines), i.e. $F_{\text{single\_critical}}\left(\omega\right)$ which is found by setting $\gamma_{i1}=\gamma_{c1}$ in Eq. (S2)

$F_{\text{single\_critical}}\left(\omega\right)=\left|\frac{\sqrt{\gamma_{\text{i1}}}}{i\Delta_{1}-\gamma_{i1}}\right|^{2}FSR_{1}$. (S11)

where $\Delta_{1}=\omega-\omega_{1}$. When $\omega=\omega_{1}$, Eq. (S11) becomes

$F_{\text{single\_critical}}\left(\omega_{1}\right)=F_{\max}=\frac{FSR_{1}}{\gamma_{i1}}$. (S12)

The $\gamma_{i1}$ used for the Eq. (S11) were converted from the corresponding $\sigma_{1}$. Comparing the spectra $F_{\text{single\_critical}}\left(\omega\right)$ (dashed red lines) and TMM (solid blue lines) results with signal bandwidth between 10 - 50 GHz, the relative percentage difference of the maximum intensity enhancement obtained by different methods is within 3.5% (**Fig. S7**). The provided spectra indicate that the pump resonance operates at a near-critical coupling condition over a wide range of design bandwidth.


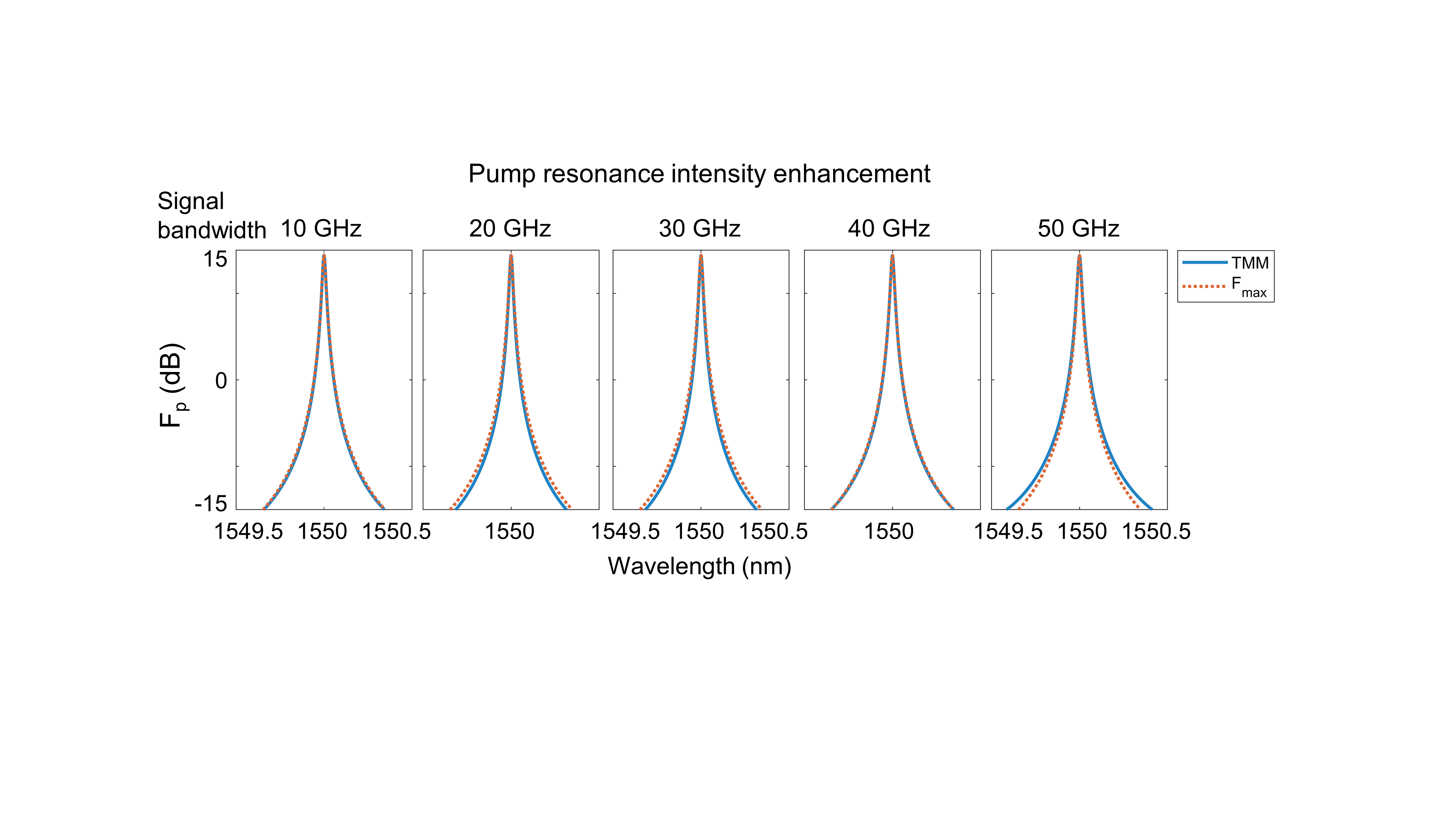


**Fig. S7 | Comparison results between the intensity enhancement spectra of the pump wave in the main resonator using TMM (solid blue lines) and the spectra of a single cavity under critical coupling using $F_{\text{single\_critical}}\left(\omega\right)$(dashed red lines) at various design signal bandwidths**.

As an alternative perspective, the TCMT offers a clearer physical picture compared to TMM. Therefore, we discuss the critical coupling condition for the pump light using the intensity enhancement derived from the TCMT, i.e., Eq. (S8). Applying zero-detuning condition ($\Delta_{1}=\Delta_{2}=0$) and critical coupling condition ($T=0$) in Eq. (S8) for simplicity, the intensity enhancement at pump frequency in the main cavity reaches maximum at

$\gamma_{c\_\max}=\frac{4g^{2}}{\gamma_{i1}}+\gamma_{i1}$ (S13)

and the corresponding maximum intensity enhancement is given by

$F_{\max}^{\text{dual}}=\frac{FSR_{1}}{\gamma_{i1}}$. (S14)

Comparing Eq. (S14) with Eq. (S12), the maximum intensity enhancement achieved in a dual-coupled cavity is identical to that in the critically coupled single cavity, i.e., $F_{\max}^{\text{dual}}=F_{\max}$. Note that Eq. (S13) also agrees with the critical coupling condition discussed in Supplementary Reference^5^. The critical coupling condition at the pump resonance can also be understood from the eigenvalue of the coupled system, as shown in **Fig. S8**. The critical coupling corresponds to the point where the low-loss branch of the imaginary part of the eigenvalues (Eq. (S6)) equals twice the intrinsic loss of the main resonator ($\gamma_{i1}/2$). This implies that the main resonator-localized (low-loss branch) pump eigenmode has an external coupling loss equivalent to the intrinsic loss of the main resonator. The condition can be written as

$Im\left(\omega_{+}\right)=-\frac{1}{4}\left(2\gamma_{i1}+\gamma_{c}\right)+\frac{1}{4}\sqrt{\gamma_{c}^{2}-16g^{2}}=-2\cdot\frac{\gamma_{i1}}{2}=-\gamma_{i1}$ , (S15)

which yields an expression simplified as $\gamma_{c\_\max}=4g^{2}/\gamma_{i1}+\gamma_{i1}$ (with an approximation of $\gamma_{i1}=\gamma_{i2}$).


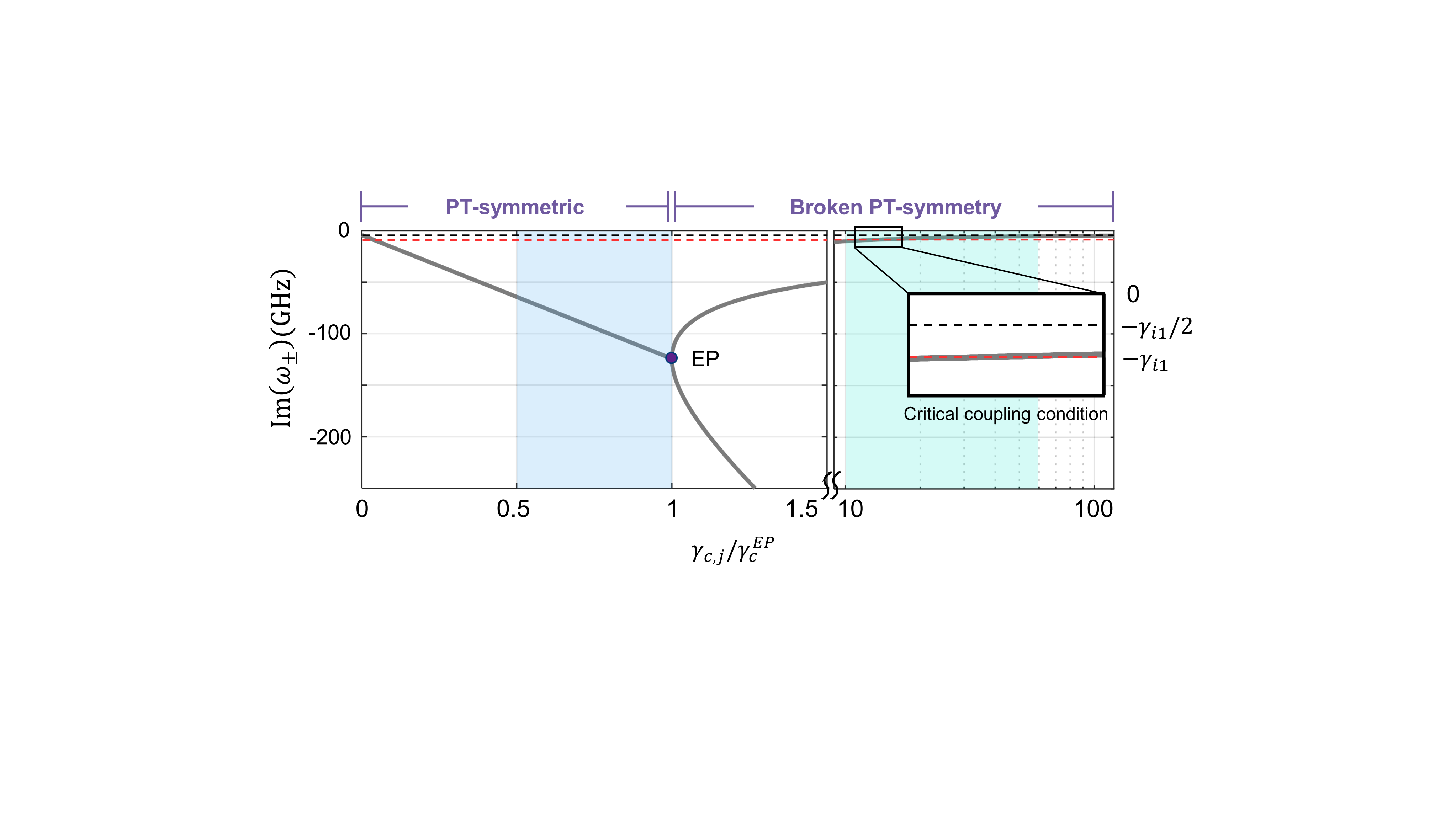


**Fig. S8 | Illustration of the critical coupling condition of the pump light from the PT-symmetry breaking point of view.** Evolution of the imaginary parts of the eigenvalues of a passive PT symmetry system as a function of the decay rate of the auxiliary cavity. Black and red dashed lines correspond to the intrinsic loss of the main cavity and twice the intrinsic loss of the main cavity, respectively.

As discussed in the main text, the destructive interference at $\omega_{p}$ gives additional loss to the pump wave on top of the coupling decay $\gamma_{c}$offered by the bus waveguide. Equation (S13) shows that $\gamma_{c\_\max}>>g$ as well as $\gamma_{c\_\max}>>\gamma_{c}$ since $g>>\gamma_{i1}$. A relationship between $\gamma_{c}$ and the total decay rate of the pump light in the effective high-loss resonator, $\gamma_{c}'$,

$\gamma_{c}'=4\frac{\left(\gamma_{c}^{2}+4FSR_{1}^{2}\right)^{2}}{\gamma_{c}\cdot\left(FSR_{1}+\frac{\left(\gamma_{c}+\gamma_{i2}\right)^{2}}{4FSR_{1}}\right)^{2}}$ (S16)

is found to be a good approximation over a wide range of $\gamma_{c}$. By substituting Eq. (S16) as $\gamma_{c}$ into Eq. (S8), the intensity enhancement of the pump light can be derived from the TCMT. **Figure S9** compares the intensity enhancement of the pump resonance obtained with different methods, where the blue and red lines represent the intensity enhancements calculated by TMM and TCMT, respectively. Good consistency is found between the results of the two methods. Note that the formal derivation of Eq. (S16) requires further studies, which is beyond the scope of this work.


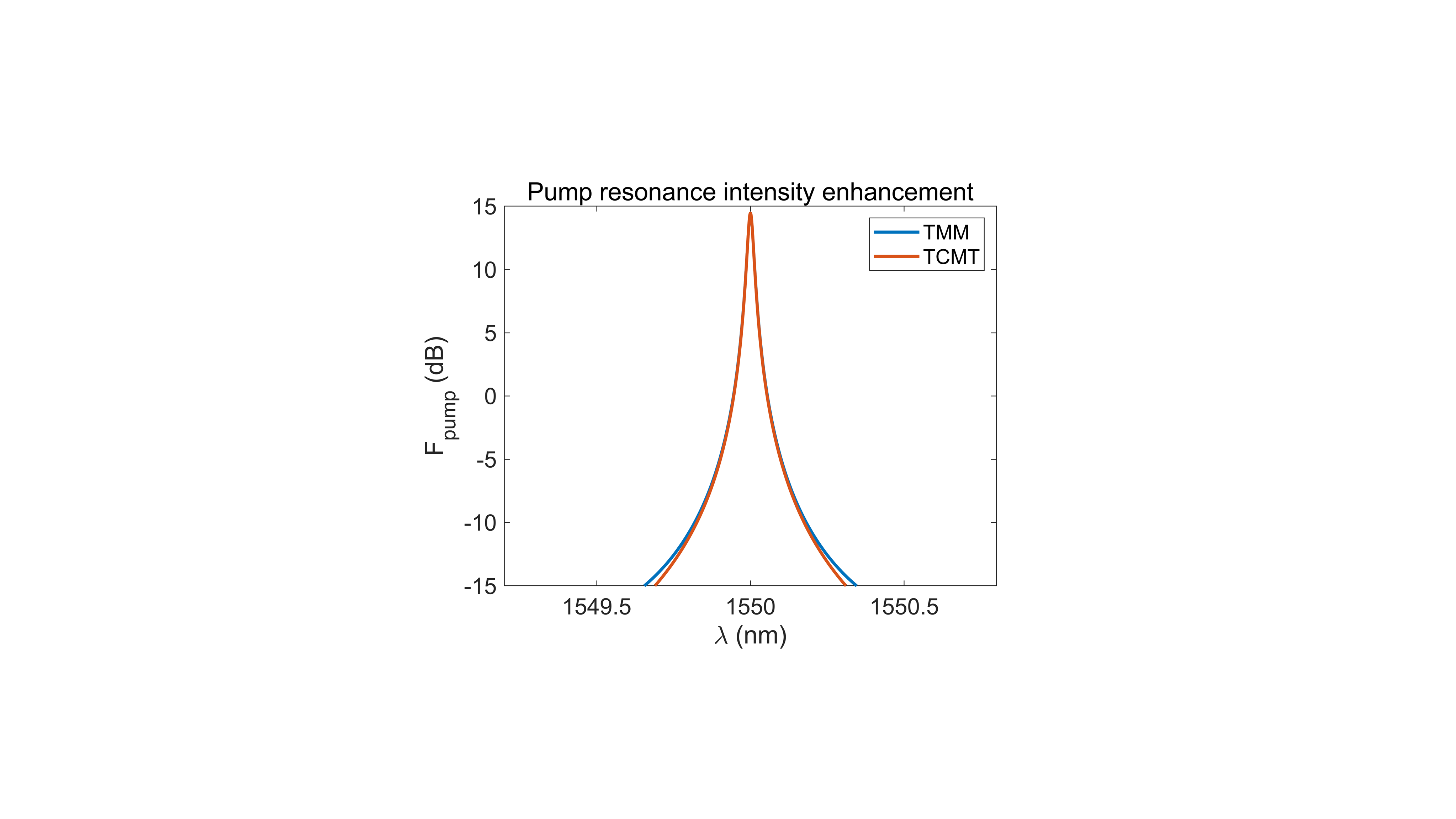


**Fig. S9 | The comparison results of the intensity enhancement spectrum between the TCMT and TMM in the main resonator** **at the pump resonance**. Parameters for the TMM coupling coefficient are$k_{2}$=0.3984, and $k_{3}$=0.7387. Parameters for the TCMT coupling decay are$g$= 138 GHz, $\gamma_{c}$= 387 GHz, and $\gamma_{c}'$= 9949.8 GHz, which is derived from Eq. (S16). The intrinsic decay rate of the main and auxiliary resonators used in the two different methods are the same, with a value of 8.75 GHz.

We further compare the intensity enhancement spectrum of the pump resonance over a wide signal bandwidth using Eq. (S16) (**Fig. S10**). The $g$ and $\gamma_{c}$ used for the TCMT analysis were converted from the corresponding $k_{2}$ and $k_{3}$. Good consistency has been found between two methods over a wide signal bandwidth.


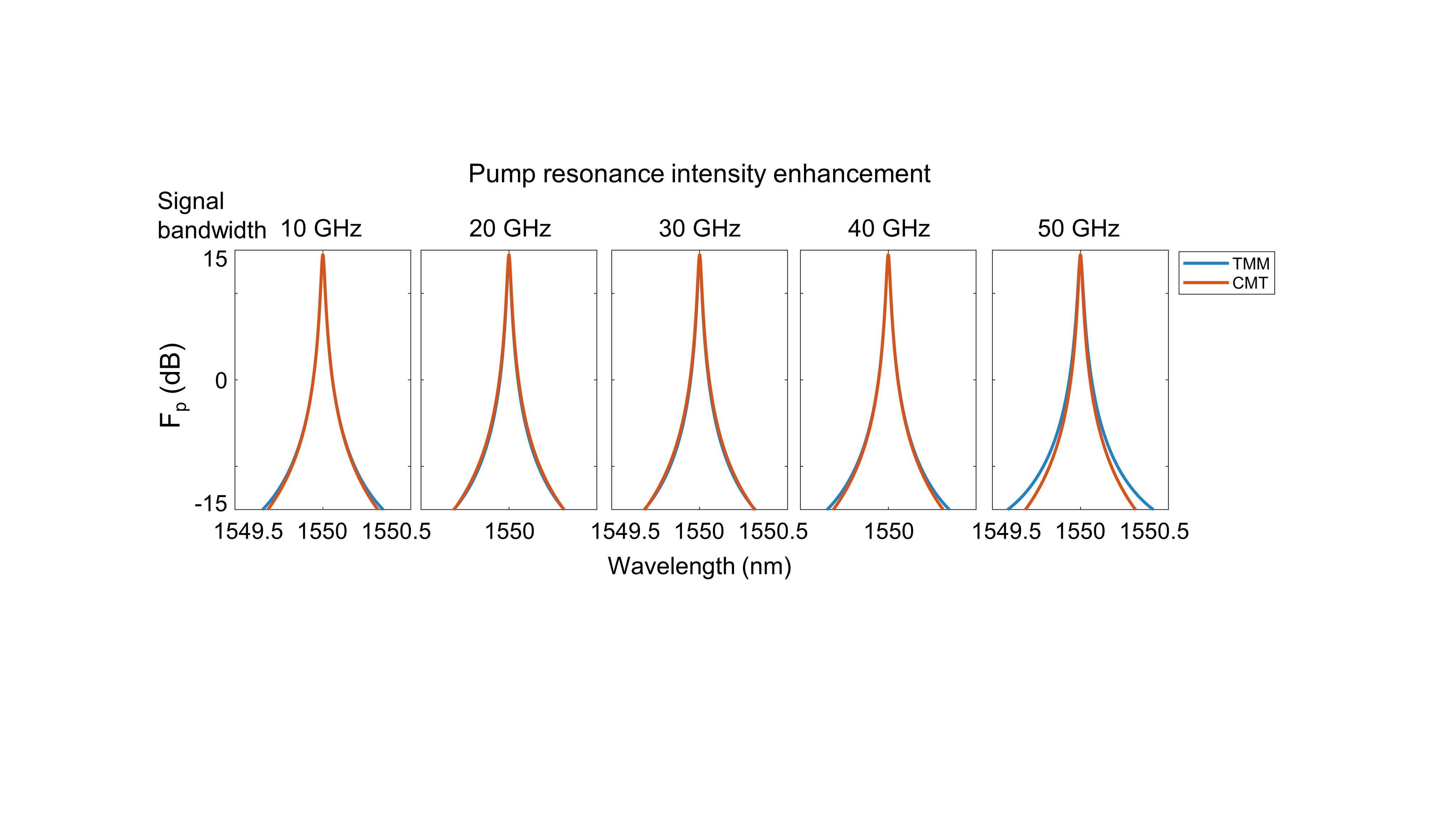


**Fig. S10 | Comparison results of intensity enhancement spectrum between the TMM and TCMT of the pump wave in the main resonator when different signal bandwidths are realized.** Blue and red lines show that the intensity enhancement spectra calculated by TMM, and TCMT, respectively.

# S4. Analytical model of FWM conversion efficiency

**Toy model**

We introduce a toy model to conveniently (1) compare the conversion efficiency of the single cavity and the dual-coupled cavities and (2) find the enhancement of the conversion efficiency when using the dual-coupled cavity system.

For the single resonator, the small-signal conversion efficiency^4^ is given by $\eta=\left|\gamma PL_{eff}\right|^{2}F_{p}^{2}F_{\mathrm{s}}F_{i}$. According to Eq. (S2) and considering the condition $\Delta_{1}=0$, the intensity enhancements $F_{p,s,i}$ for the pump (*p*), signal (*s*), and idler (*i*) waves are the same and can be expressed as:

$F_{p,s,i}=\frac{4\gamma_{c1}FSR_{1}}{\left(\gamma_{i1}+\gamma_{c1}\right)^{2}}$. (S17)

The maximum intensity enhancement is achieved at a critical coupling condition$\gamma_{i1}=\gamma_{c1}$:

$F_{\max}=\frac{FSR_{1}}{\gamma_{i1}}$, (S18)

which is the same as Eq. (S12). When the resonance linewidth of a resonator is narrower than the bandwidth $B$of a high-speed optical signal, the resonator is generally over-coupled ($\gamma_{c1}>>\gamma_{i1}$) to satisfy the linewidth requirement $\Delta\omega=\gamma_{i1}+\gamma_{c1}\approx\gamma_{c1}=2\pi B$. In this case, the intensity enhancement is given by $F_{p,s,i}$=$2FSR_{1}/\pi B$, and subsequently, the conversion efficiency of the single cavity case can be expressed as

$\eta=\left|\gamma PL_{eff}\right|^{2}F_{s}^{4}=\frac{16\left|\gamma PL_{eff}\right|^{2}FSR_{1}^{4}}{\left(\pi B\right)^{4}}$. (S19)

Now we consider the case for the toy model mentioned in the main text for the dual-coupled cavity case where the linewidth of the resonance for the pump wave is selectively reduced to achieve maximum intensity enhancement $F_{\max}$ (Eq. S19) while that of the signal and idler $F_{s,i}$ remains the same as the single cavity case. The conversion efficiency of this configuration can be written as

$\begin{matrix} \eta=\left|\gamma PL_{eff}\right|^{2}F_{\max}^{2}F_{s}^{2}=\left|\gamma PL_{eff}\right|^{2}\left(\frac{FSR_{1}}{\gamma_{i1}}\right)^{2}\left(\frac{2FSR_{1}}{\pi B}\right)^{2} \\ \text{ }=\frac{4\left|\gamma PL_{eff}\right|^{2}FSR_{1}^{4}}{\left(\gamma_{i1}\pi B\right)^{2}} \end{matrix}$ (S20)

It is evident that $\eta$ are proportional to $B^{-4}$ and $B^{-2}$ for the single cavity and the dual-coupled cavities cases, respectively. The enhancement of conversion efficiency $G$, defined as $\eta$ between our design and the single cavity device designed for the same $B$, can thus be derived from Eq. (S20) and Eq. (S21) as

$G=\left(\frac{F_{\max}}{F_{B}}\right)^{2}\approx\left(\frac{\pi B}{2\gamma_{i1}}\right)^{2}$. (S21)

**Analytical conversion efficiency based on TMM and nonlinear Schrödinger equations**

For the conversion efficiency of the dual cavity system, we show the derivation of the FWM conversion efficiency based on the TMM and nonlinear Schrödinger equations. It is known that $\eta$ is proportional to $F_{p}^{2}F_{s}F_{i}$ in the single cavity case^4^. In the following, we show such a relation is also valid in the dual-coupled cavity design by following the procedure described in Supplementary Ref.^4^.

According to the TMM, $\eta=\left|\frac{E_{out\_i}}{E_{in\_s}}\right|^{2}$, which can be further expanded as

$\eta=\left|\frac{E_{out\_i}}{E_{in\_s}}\right|^{2}=\left|\frac{E_{out\_i}}{E_{t2\_i}}\cdot\frac{E_{t2\_i}}{E_{i2\_s}}\cdot\frac{E_{i2\_s}}{E_{in\_s}}\right|^{2}=F_{main\_\mathrm{s}}\left|\frac{E_{out\_i}}{E_{t2\_i}}\cdot\frac{E_{t2\_i}}{E_{i2\_s}}\right|^{2}$ , (S22)

where $E_{t2\_i}$ is the idler generated inside the main cavity, which can be found by using the nonlinear Schrödinger equations,

$\begin{matrix} \frac{\partial E_{p}}{\partial z}=-\frac{\alpha_{1}}{2}E_{p}\text{ , }\frac{\partial E_{s}}{\partial z}=-\frac{\alpha_{1}}{2}E_{s}\text{ ,} \\ \frac{\partial E_{i}}{\partial z}=-\frac{\alpha_{1}}{2}E_{i}+i\gamma E_{p}^{2}E_{s}^{*}\exp\left(i\Delta kz\right), \end{matrix}$ (S23)

where $\gamma$ is the effective nonlinearity, $\alpha_{1}$is the propagation loss, is the phase mismatch factor defined by$\Delta k=2k_{p}-k_{s}-k_{i}$ , and $k_{p,s,i}$ are the propagation constants of the pump (*p*), signal (*s*), and idler (*i*), respectively. Applying the relationship between $\alpha_{1}$and roundtrip field loss $\sigma_{1}$, we have $\sigma_{1}=\exp(-\frac{\alpha_{1}L_{1}}{2})$. Therefore, $E_{t2\_i}$ is found to be

(S24)

where $P=\left|E_{in\_p}\right|^{2}$ is the pump power coupled to the bus waveguide, $L_{eff}$ is the effective length defined by $L_{eff}^{2}=L_{1}^{2}\cdot\exp(-\alpha_{1}L_{1})\cdot\left|\frac{1-\exp\left(-\alpha_{1}L_{1}+i\Delta kL_{1}\right)}{\alpha_{1}L_{1}-i\Delta kL_{1}}\right|^{2}$, $L_{1}$ is the length of the main cavity, and $F_{main\_p}=\left|\frac{E_{i2\_p}}{E_{in\_p}}\right|^{2}$is the intensity enhancement of pump light in the main cavity. For $E_{out\_i}$ we have：

(S25)

Finally, we obtain *η* as:

(S26)

We note that various combinations of the $k_{2}$ and $k_{3}$ in the dual cavity systems result in the same bandwidth. The conversion efficiency - bandwidth relationship of the dual cavity presented in **Fig. 3d** (main text) is obtained by taking the maximum conversion efficiency (from the TMM) at a given FWM bandwidth (determined by different combinations of the $k_{2}$ and $k_{3}$).

# S5. Full-map coupled nonlinear Schrödinger equations

In order to track the four-wave mixing (FWM) process – generation of the idler wave – in the PT-symmetric device and accurately estimate the nonlinear efficiency, we employ a rigorous nonlinear model based on a series of full-map coupled nonlinear Schrödinger equations^3^. The coupled equation is written as:

(S27)

where is the circular angle, $T$ is the time; *L_main_* and *L_aux_* are the length of the main cavity (*main*) and the auxiliary cavity (*aux*), $E_{main},E_{aux}$ are the complex intracavity field, $E_{in}$ is the input field amplitude, $k_{main},k_{aux}$ are the wave vectors, $k_{main}^{'}=\left.dk_{main}/d\omega\right|_{\omega=\omega_{p}},k_{aux}^{'}=\left.dk_{aux}/d\omega\right|_{\omega=\omega_{p}}$are the reciprocals of the group velocities at the pump angular frequency$\omega_{p}$,are the group velocity dispersions, and $\delta$ is the Dirac delta function, are the intrinsic amplitude losses per unit length, are the nonlinear coefficients of the main cavity and the auxiliary cavity.

The full-map model can be used to verify both the linear and nonlinear behavior of the system. By setting the nonlinear coefficient $\gamma$ to zero, the model is completely linear and agrees well with the transmission spectrum as well as intensity enhancement given by the TMM (**Fig. S11**).


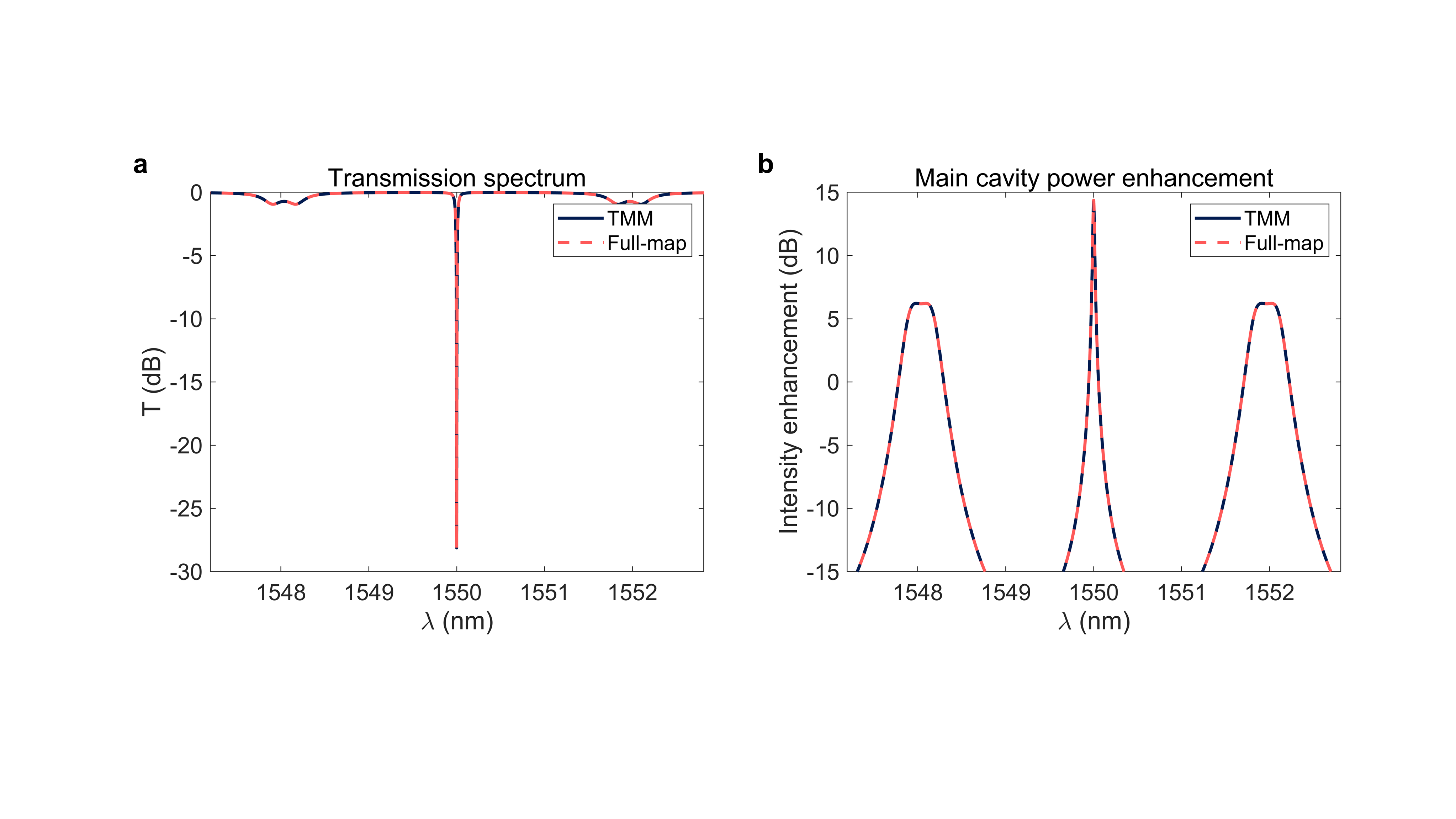


**Fig. S11 |** **Comparison of full-map model and TMM. a**, Transmission spectrum of the system. **b**, Intensity enhancement of the main cavity.

Note that, as a consequence of the 2:1 ratio between the length of the main and auxiliary cavities, the transmission spectrum depicted in **Fig. S11a** will repeat over the wavelength (resonances alternating with broad and narrow linewidths). This is evident from the experimental transmission spectrum illustrated in **Fig. S12**.


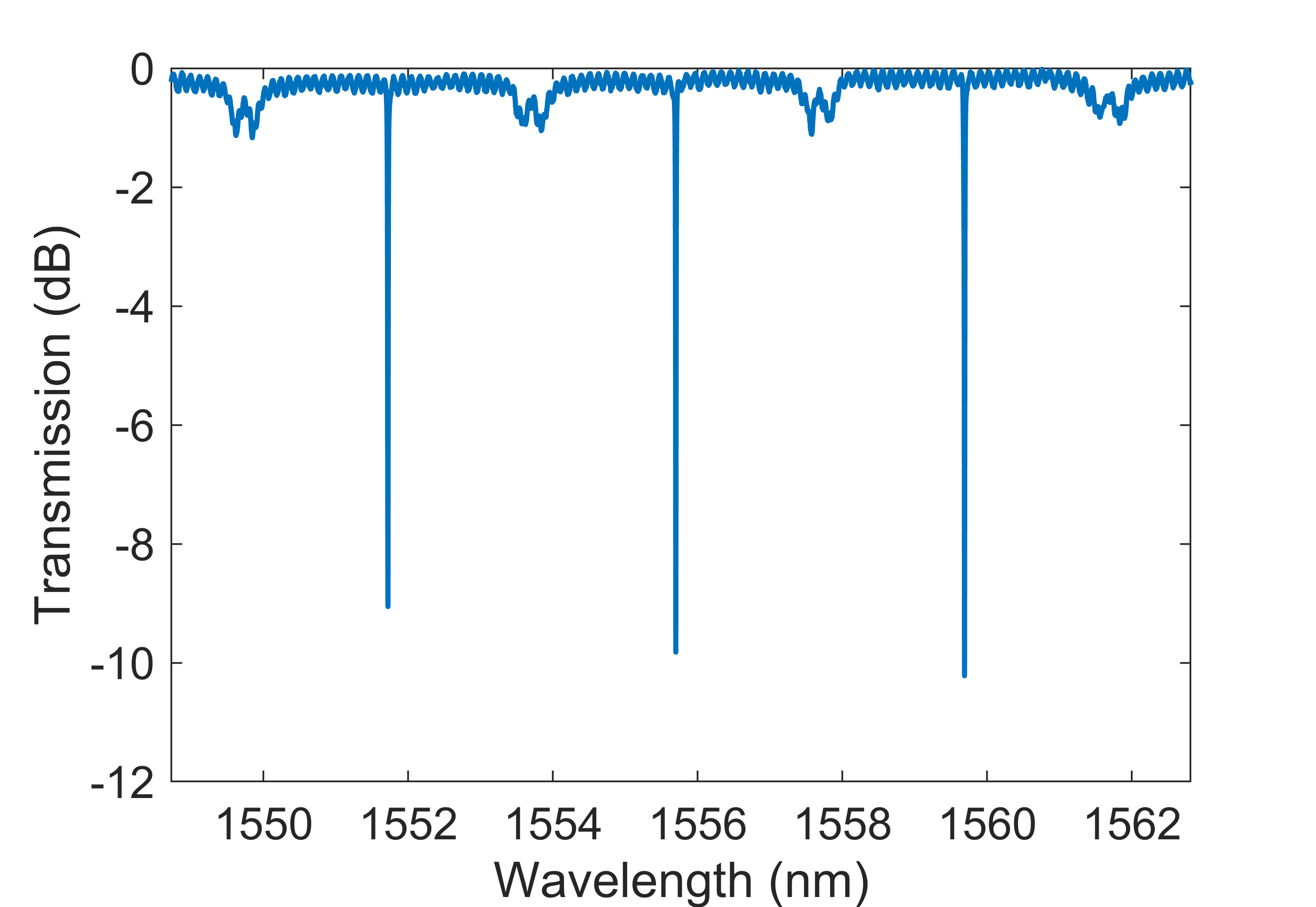


**Fig. S12 |** **Transmission measured from dual-coupled cavity system.**

**Figure S13** compares the nonlinear behavior of the system, i.e., the conversion efficiency of the resonator systems derived by the TMM that follows Eq. (S27) (identical to the solid purple line in **Fig. 3d** in the main text) and the full map method described in Eq. (S27). The two methods show excellent agreement with each other and are experimentally validated in **Fig. 3d**.


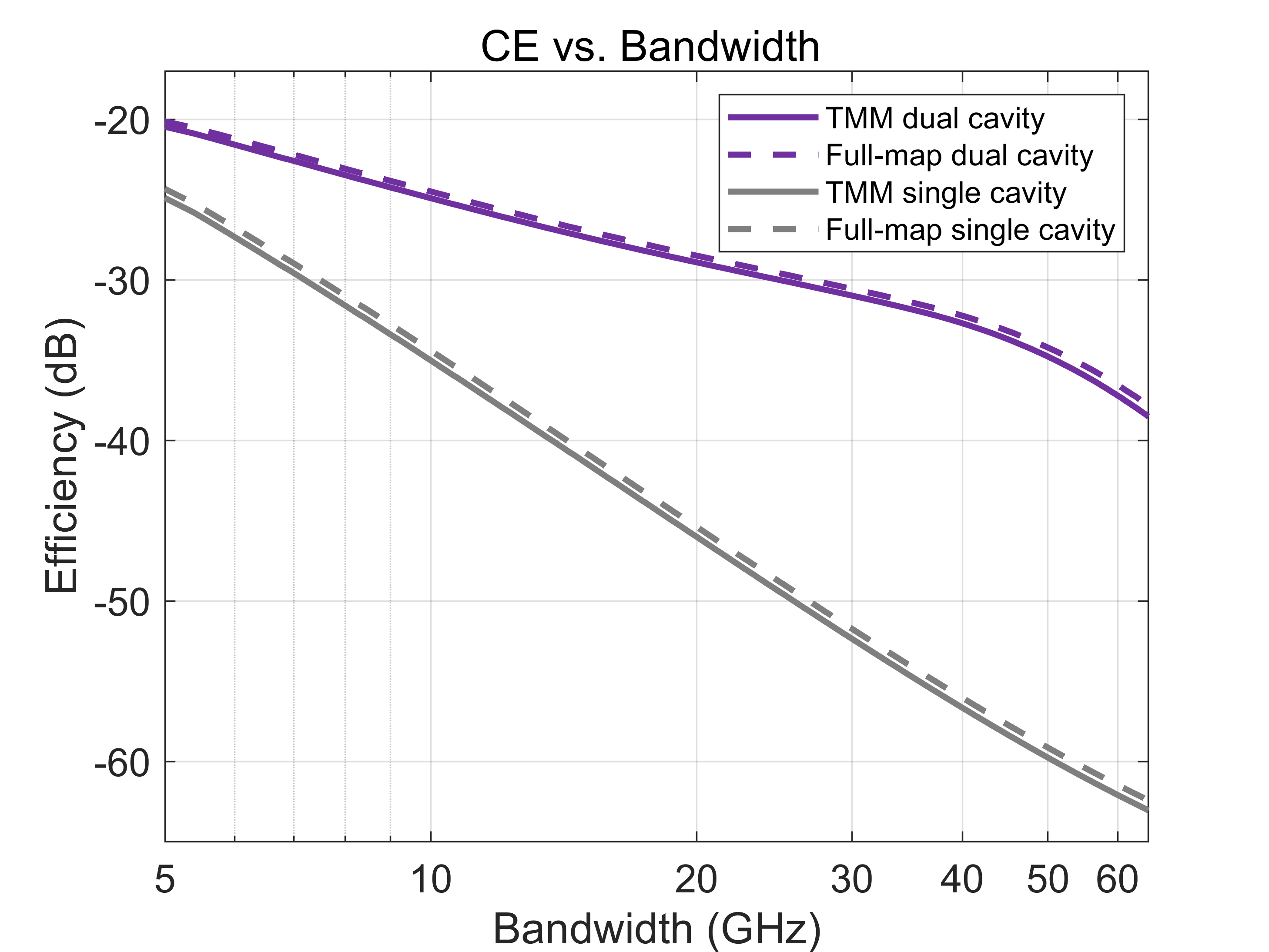


**Fig. S13 | Comparison between conversion efficiency derived by TMM and full-map model.** Solid purple and grey lines: the conversion efficiencies at varying FWM bandwidths, calculated by TMM of dual and single cavity systems, respectively. Dashed purple and grey lines: conversion efficiencies at varying FWM bandwidths, calculated by full-map equations of dual and single cavity systems, respectively.

# S6. Synthetic linewidth of dual cavity

The linewidth of dual-coupled resonator systems can be represented by synthetic linewidth, which is determined by the profile of the hybridized resonance. By exploiting the features of the real and imaginary parts of the system eigenvalues, the synthetic linewidth$\Delta\omega$ of the coupled system can be conveniently expressed as

$\Delta\omega=\mathrm{Re}\left(\omega_{+}\right)-\mathrm{Re}\left(\omega_{-}\right)+2\mathrm{Im}\left(\omega_{+}\right)$, (S28)

which incorporates the mode splitting and total loss of the supermodes. **Figure S14** shows the comparison between the 3-dB bandwidth of the normalized transmission spectrum (purple lines) and the synthetic linewidth of the system derived by Eq. (S28) (red lines), where the good consistency between the results confirms the reliability of Eq. (S28). Note that Eq. (S28) is only valid for the area close to the EP and broken PT-symmetry regime. When the excessive splitting feature (3-dB center shallowing with respect to the transmission minima) appears, the system transmission spectrum splits into two resonances with the same linewidth $\Delta\omega=2\mathrm{Im}\left(\omega_{+}\right)$.


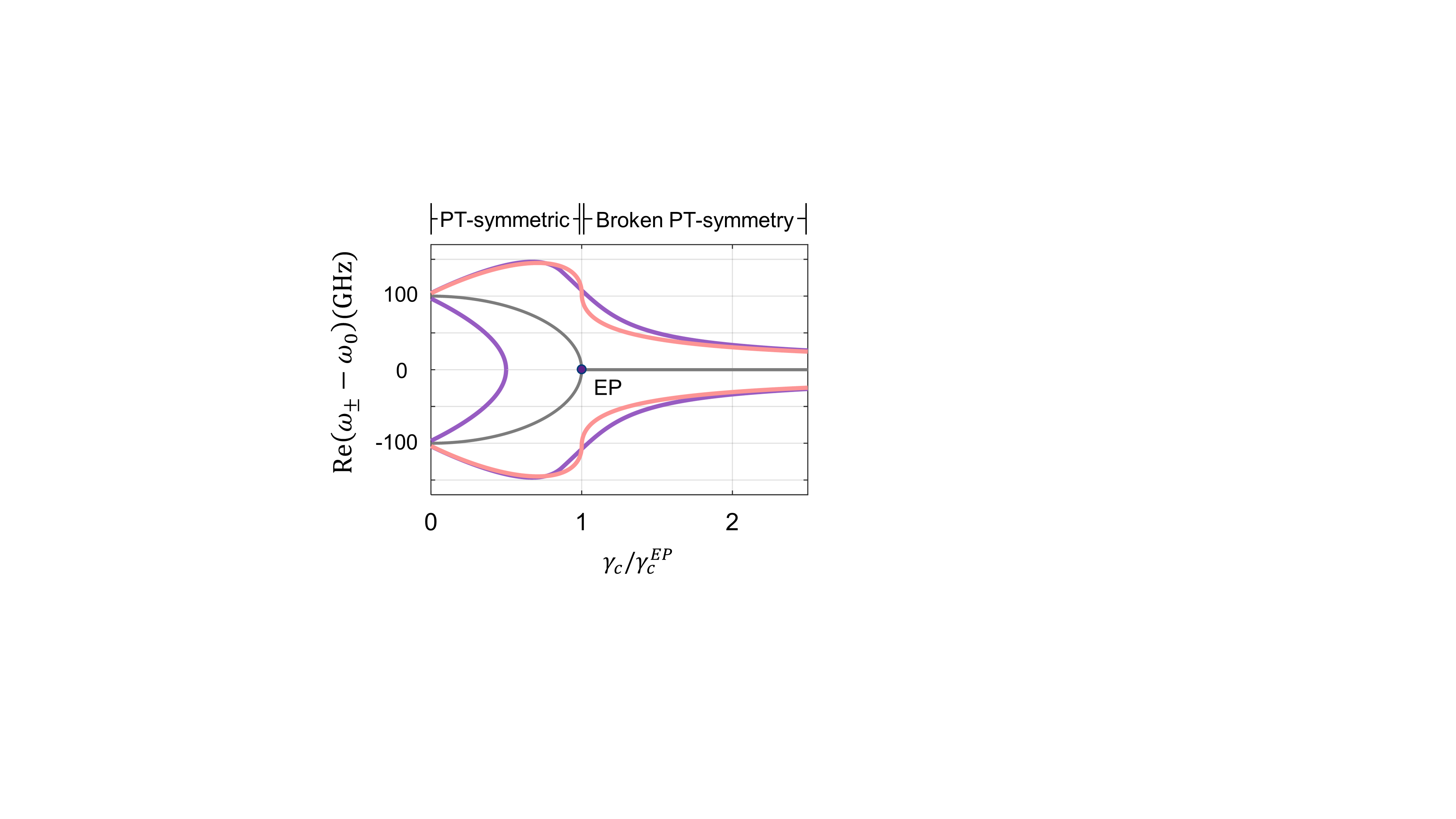


**Fig. S14 | Evolution of the** **system synthetic linewidth with varying** $\gamma_{c}/\gamma_{c}^{EP}$**.** Purple lines represent the relative frequencies at which the normalized transmission becomes 1/2 at a given $\gamma_{c}/\gamma_{c}^{EP}$; the spectral distance between red lines indicates the synthetic linewidth of the system given by Eq. (S28) at a given $\gamma_{c}/\gamma_{c}^{EP}$.

# S7. Design guideline

Equation (S27) gives an accurate estimation of the small-signal conversion efficiency of the dual-coupled cavity structure. We scan the$k_{2}$ and $k_{3}$ to acquire the enhancement of conversion efficiency (**Fig. S15a**), which is related to the $G$ defined in Eq. (1) in the main text. The calculation of the conversion efficiency and signal bandwidth follow the definition given in the main text (**Fig. 3c**). The white area corresponds to the PT-symmetric regime that is unsuitable for signal processing (excessive splitting in the resonance profile – with more than 3-dB center shallowing with respect to the transmission minima). We note that the coupled resonator can be designed for a specific signal bandwidth with different combinations of $k_{2}$ and $k_{3}$ according to **Fig. S15b**. The solid purple line in **Fig. 3d** (main text) is extracted by taking the maximum conversion efficiency at a given signal bandwidth.


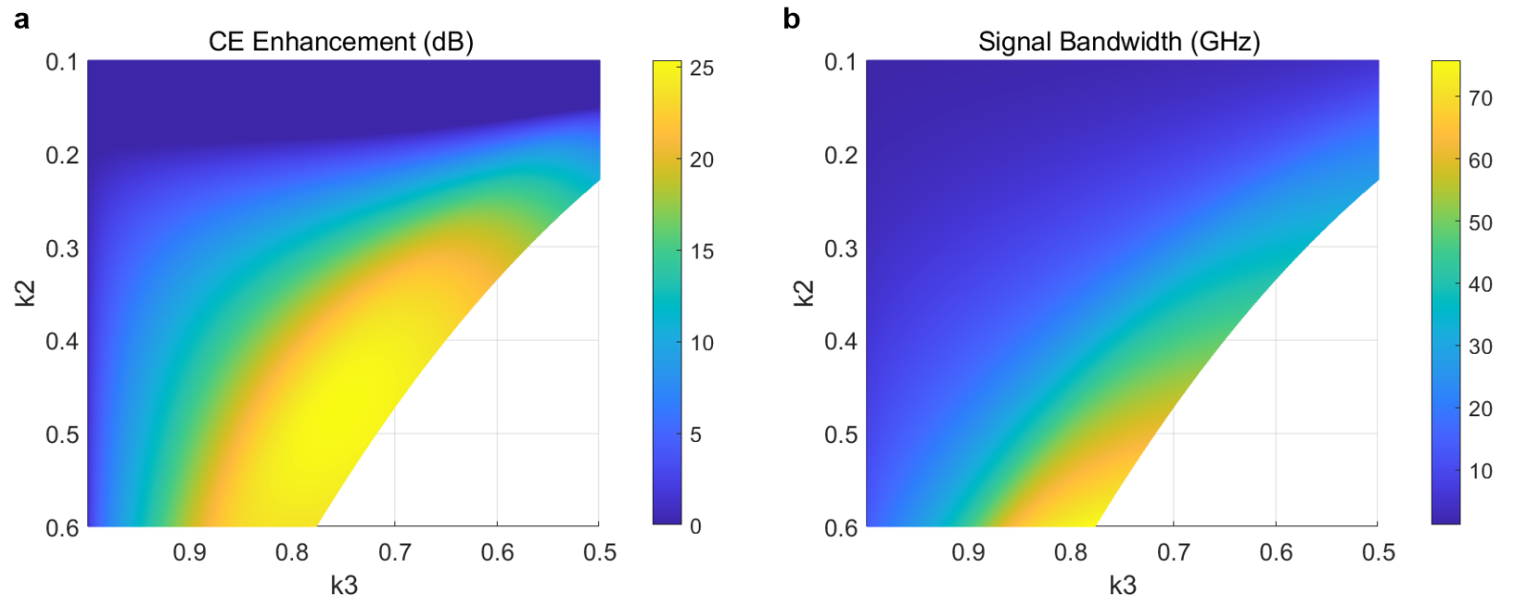


**Fig. S15 | Performance comparison of a coupled resonator and an all-pass single microresonator system. a,** Enhancement of conversion efficiency and **b,** signal bandwidth of the coupled resonator system corresponding to $k_{2}$ and $k_{3}$**.** Conversion efficiency enhancement values equal to or lower than 0 dB are colored the same in **a**.

**Figure S16** is provided to aid in designing an optimal device for a given signal bandwidth by combining the information given in **Fig. S15a, b**. The grey lines indicate the contour lines of the signal bandwidth based on **Fig. S15b**. The dashed purple line corresponds to the EP, which is given as a rough guideline for device design, as mentioned in the main text. The area within the red lines indicates the condition in which the signal resonance is operating in the PT-symmetric regime without the excessive splitting feature (3-dB center shallowing with respect to the transmission minima). The area within the blue lines represents the transmission extinction ratio of the pump resonance being lower than -10 dB (according to Eq. (S9)), thus indicating the near-critical coupling condition of the pump resonance. The intersection of the red and blue regions indicates the optimal operation region which covers a wide range of signal bandwidths from 30 GHz to 60 GHz. We highlight that the conversion efficiency enhancement beyond 20 dB is achieved within a large parameter space (overlapping area of the red and blue region) of $k_{2}$ and $k_{3}$ – covering the signal bandwidth from 30 GHz to 70 GHz. We show that the coupled resonator can be optimally designed with a wide range of coupling parameters.


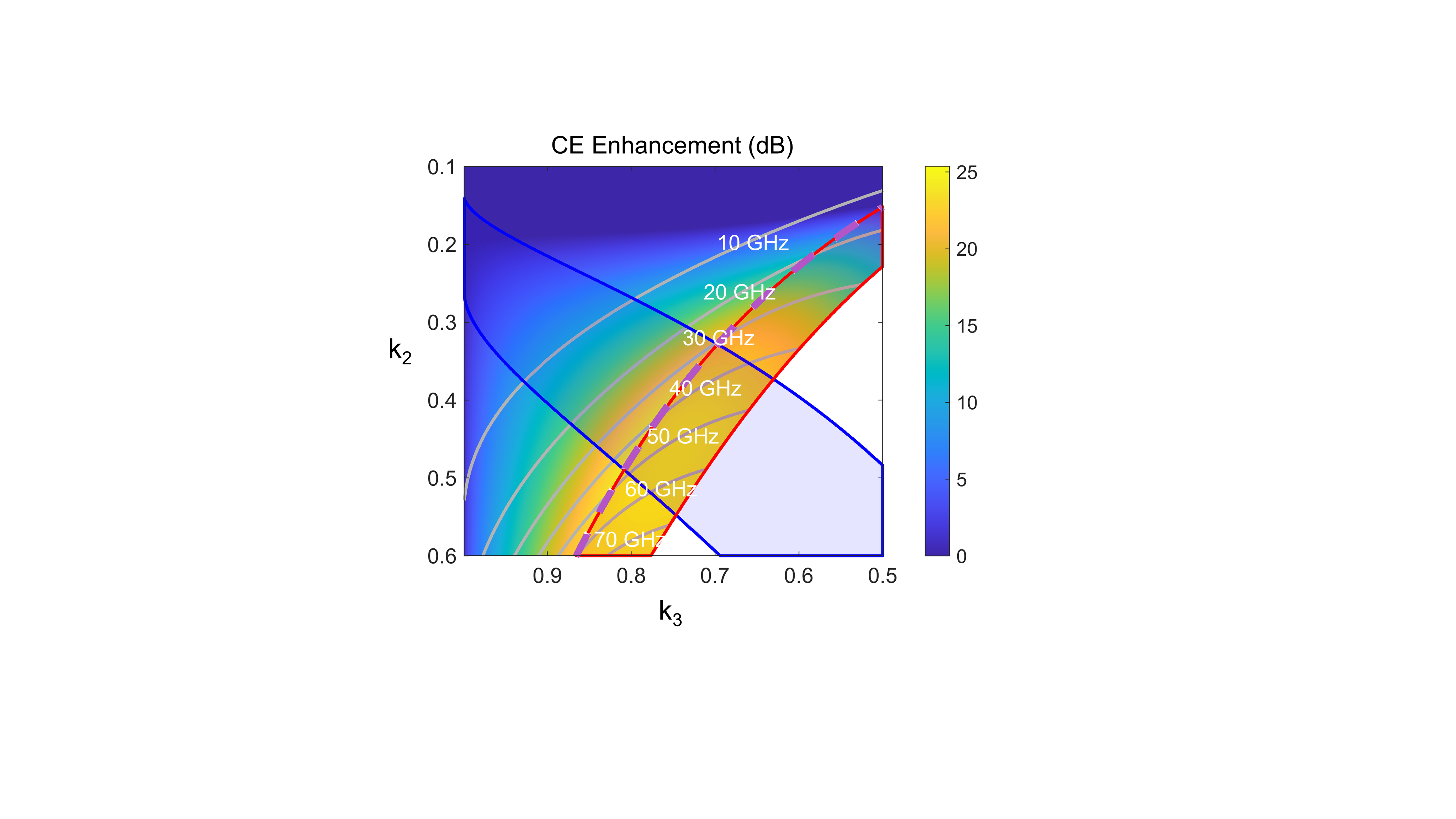


**Fig. S16 | Design guideline of the PT symmetry system.** The colormap displays the enhancement of the conversion efficiency (CE) in our design as a function of the coupling coefficients ($k_{2}$ and $k_{3}$). The coupled resonator is compared to the all-pass microresonator designed for the same signal bandwidth. The regions inside the blue and red lines represent the optimum parameter range for the pump wave and signal/idler wave, respectively. The gray lines indicate the contour lines of the signal bandwidth based on **Fig. S15b**.

# S8. Intracavity field distribution


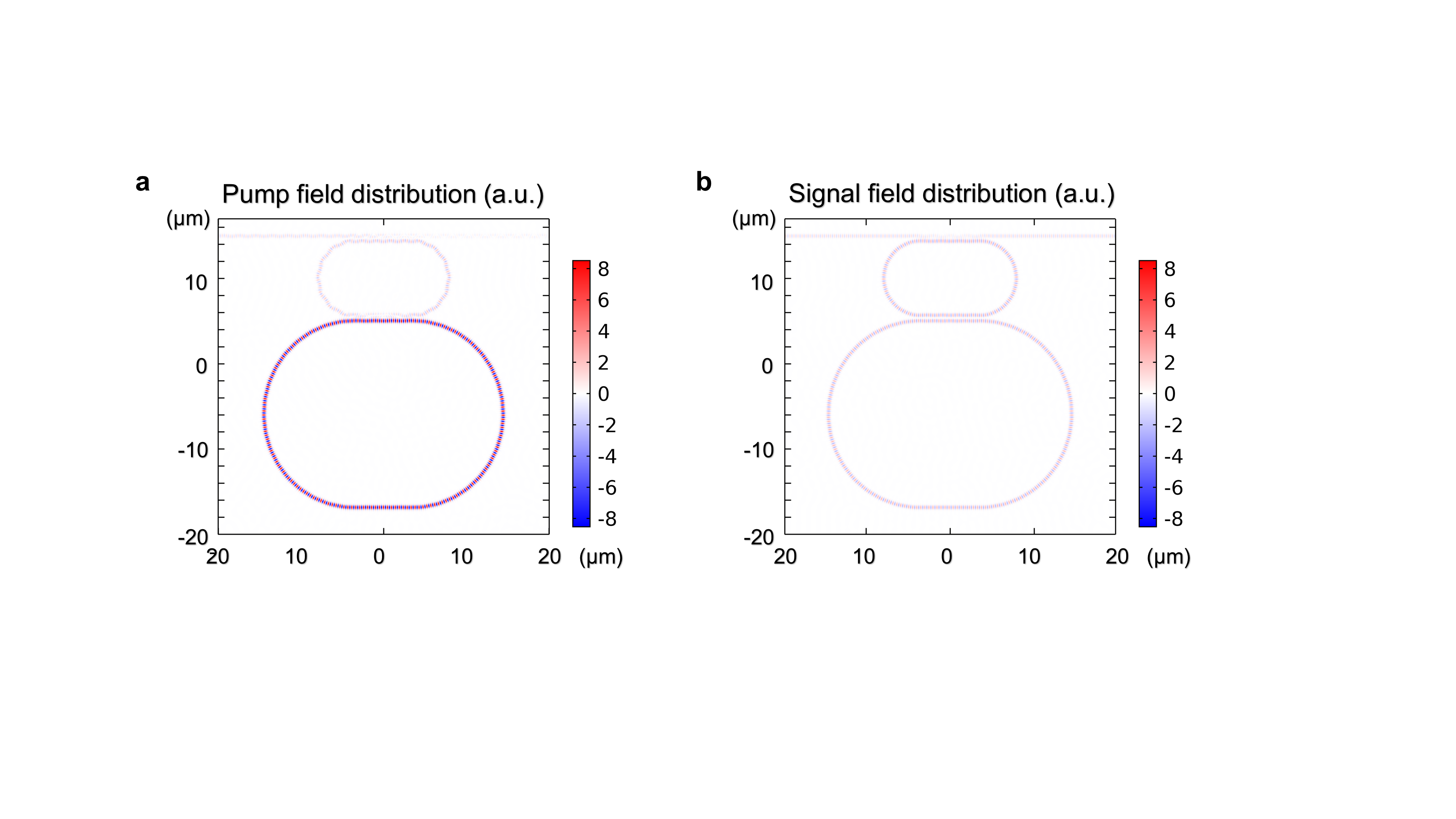


**Fig. S17 |** **Simulated** **intracavity field distribution normalized by the input field amplitude. a,** Intracavity field distribution of a pump wave. **b,** Intracavity field distribution of a signal (or idler) wave.

We use the same type of structure to reveal the intracavity field distribution through a finite element method electromagnetic wave simulation (COMSOL Multiphysics). We construct a two-dimensional model of the coupled microresonators in the simulation domain where the waveguide widths are 450 nm for the bus waveguide and the resonators. We show an optimum simulated field distribution for the pump and signal (idler) waves by implementing proper geometric configurations (resonator lengths and coupling gaps) and optical properties (excitation frequency, effective refractive index, and propagation loss). The simulated propagation loss is 5 dB/cm, and the length of the main resonator is 82.74 µm, which is twice the length of the auxiliary resonator. **Figure S17** shows the intracavity field distribution of the pump and signal waves, normalized by the input field amplitude, indicating that the intensity enhancement of the pump is noticeably higher than that of the signal wave. The intracavity field distribution shows that the signal (idler) wave circulates in both cavities while the pump is primarily confined in the main cavity, as determined by the eigenvectors at the signal and pump resonances. We also note that the pump and signal (idler) waves are spatially overlapping primarily in the main cavity. The pump field in **Fig. S17a** exhibits a field distribution resembling a high-order mode, especially in the auxiliary resonator. However, this is solely an artifact caused by a simulation convergence issue resulting from the high-Q pump resonance. The structure is designed to operate exclusively in the fundamental TE mode at any resonance (pump/signal/idler). As can be seen from the broadband transmission spectrum of the device shown in **Fig. S12**, no higher-order modes were observed.

# S9. PT-symmetry features by varying the intracavity coupling rate

To further verify the PT symmetry feature of our structure, the transmission spectra of signal/idler resonances shown in **Fig. S18** are measured experimentally by varying the coupling rate $g$ of a single device via electrothermal tuning^6^. When tuning the coupling rate by applying power to the heater labeled ‘g tuning’, the auxiliary resonance will experience a non-negligible thermal shift. As this alters the resonance alignment between the main and auxiliary resonators, we apply heat to the main resonator to compensate for the thermal shift of the auxiliary resonator and maintain the alignment. Note that the measured device has an additional waveguide coupled with the main cavity, compared with the dual cavity system. We add a note that this device can also reveal the PT features of the dual cavity system, since the waveguide coupled with the main cavity can be considered as the extra decay rate of the main cavity. The evolution of the mode splitting extracted from the transmission spectra (circles) agrees well with the TCMT predictions. We observe that as the coupling strength becomes smaller, the mode splitting gradually decreases until it vanishes when the EP is reached, verifying that the PT-symmetry is broken. Such operation also allows for modifying the intracavity resonance shape, i.e., underpinned by the resonance profile evolution from Lorentzian to a rectangular (flat-bottom) shape, which is advantageous for system performance.


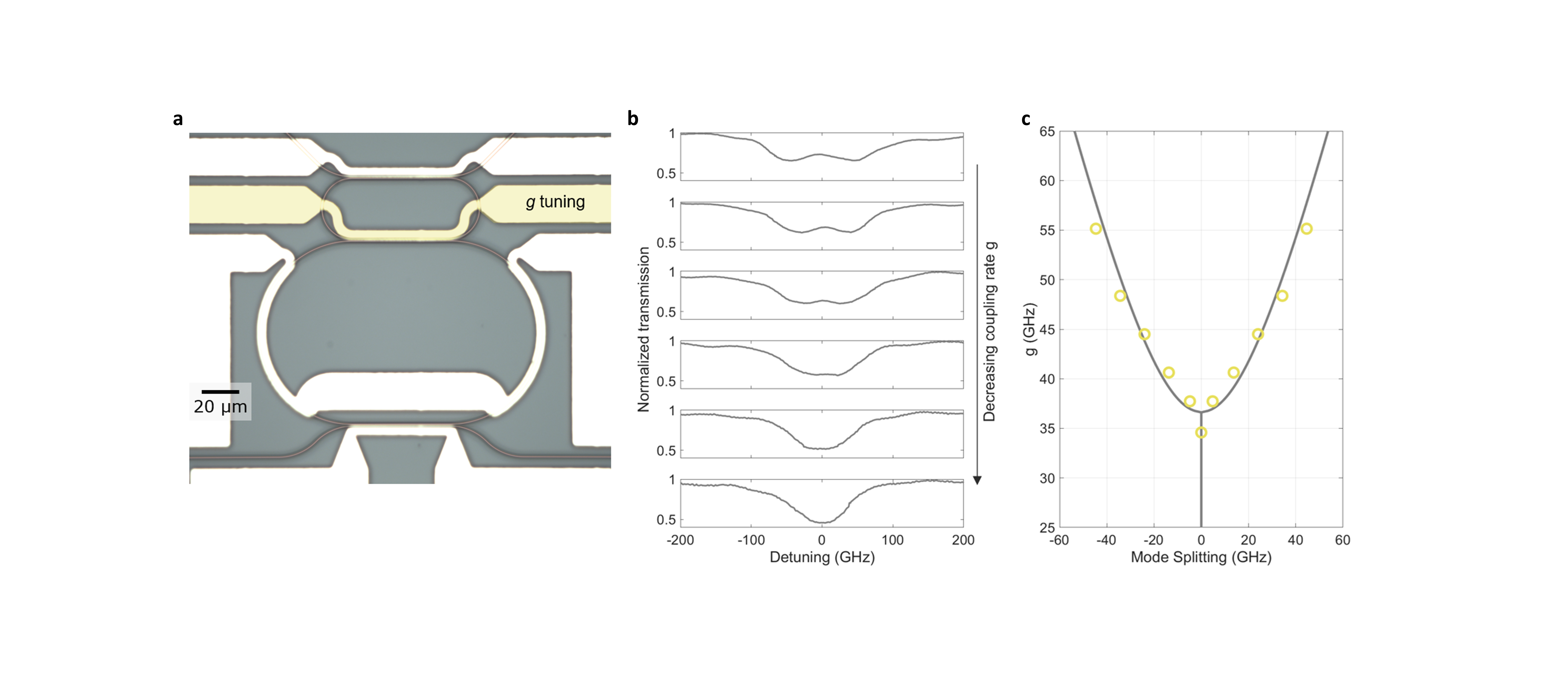


**Fig. S18 |** **Evolution of the transmission spectrum and the eigenfrequencies of signal/idler resonances as a function of coupling rate $g$. a**, Optical microscope image of the coupled resonator with microheaters. A microheater strip highlighted with yellow shade is used for tuning the coupling rate $g$ and a microheater on top of the main resonator is controlled to maintain resonance alignment. **b**, Transmission spectrum measured experimentally showing the effect of coupling rate$g$**.** **c**, Evolution of the eigenfrequencies of the supermodes at different coupling rate$g$. Circles indicate the eigenfrequencies extracted from the measured transmission spectrum. Gray lines denote the best theoretical fit to the experimental data.

Note that the coupled resonator device consists of two racetrack (straight-bend) resonators, with their straight sections being equal in length and width – forming a directional coupler. We verified the tunability of the directional coupler by using a dummy resonator (**Fig. S19a**) that has an identical dimension to the main resonator shown in **Fig. S18a**. As shown in **Fig. S19b**, the applied heat – on the bus side of the directional coupler – thermally induces phase mismatch across each arm of the directional coupler, thus reducing the coupling coefficient with applied heat. Here, the coupling coefficients are extracted from the resonance profile (transmission spectrum) at different heater power levels.


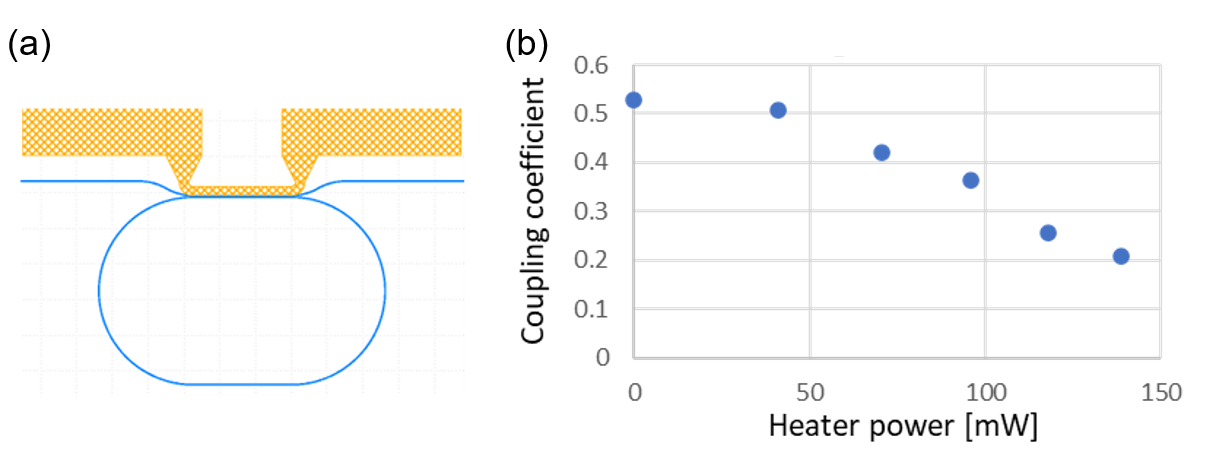


**Fig. S19 |** **Verifications of the tunability of coupling rate $g$. a**, Schematic diagram of the dummy resonator used for verifications. **b**, Extracted coupling coefficient as a function of heater power (the conversion between coupling coefficient and *g* is given in **Tab. S1**).

# References:

1. Peng, B. *et al.* Parity–time-symmetric whispering-gallery microcavities. *Nature Phys* **10**, 394–398 (2014).

2. Van, V. *Optical Microring Resonators: Theory, Techniques, and Applications*. (CRC Press, 2016).

3. Xue, X., Zheng, X. & Zhou, B. Super-efficient temporal solitons in mutually coupled optical cavities. *Nat. Photonics* **13**, 616–622 (2019).

4. Absil, P. P. *et al.* Wavelength conversion in GaAs micro-ring resonators. *Opt. Lett.* **25**, 554 (2000).

5. Komagata, K. *et al.* Dissipative Kerr solitons in a photonic dimer on both sides of exceptional point. *Commun Phys* **4**, 1–13 (2021).

6. Orlandi, P. *et al.* Tunable silicon photonics directional coupler driven by a transverse temperature gradient. *Opt. Lett., OL* **38**, 863–865 (2013).
