# Supplementary material for: Parity-time symmetry enabled ultra-efficient nonlinear optical signal processing
Source: eLight. 2024 Apr 4;4(1):6. doi: 10.1186/s43593-024-00062-w (PMC10995095; doi:10.1186/s43593-024-00062-w)
Supplement: Supplementary file 2 — Additional file 2: Extended Data Fig. S1. Performance comparison of reported all-optical wavelength conversion systems realized on integrated photonic platform. Rectangular, circular, star-shaped markers represent the type of the reported devices: waveguides, resonators, and this work, respectively. The color shading of the markers indicates the device lengths. Extended Data Fig. S2. Fabrication process of coupled resonator devices. Extended Data Fig. S3. Schematic of the wavelength conversion experimental setup. ECDL external cavity diode laser, PC polarization controller, MZM Mach-Zehnder modulator, RFA radio frequency power amplifier, BPG bit pattern generator, EDFA Erbium-doped fiber amplifier, VOA variable optical attenuator, BPF bandpass filter, CR coupled resonator device, PD photodiode, and DSO digital storage oscilloscope. Extended Data Fig. S4. FWM wavelength conversion (phase-matching) bandwidth. a Simulated group velocity dispersion (GVD) of the AlGaAsOI waveguide with a width of 465 nm and a thickness of 290 nm. b Red line is the simulated normalized conversion efficiency according to the GVD information in a, and blue circles are the conversion efficiency data points extracted from Fig. 4d. [file 43593_2024_62_MOESM2_ESM.docx]

**
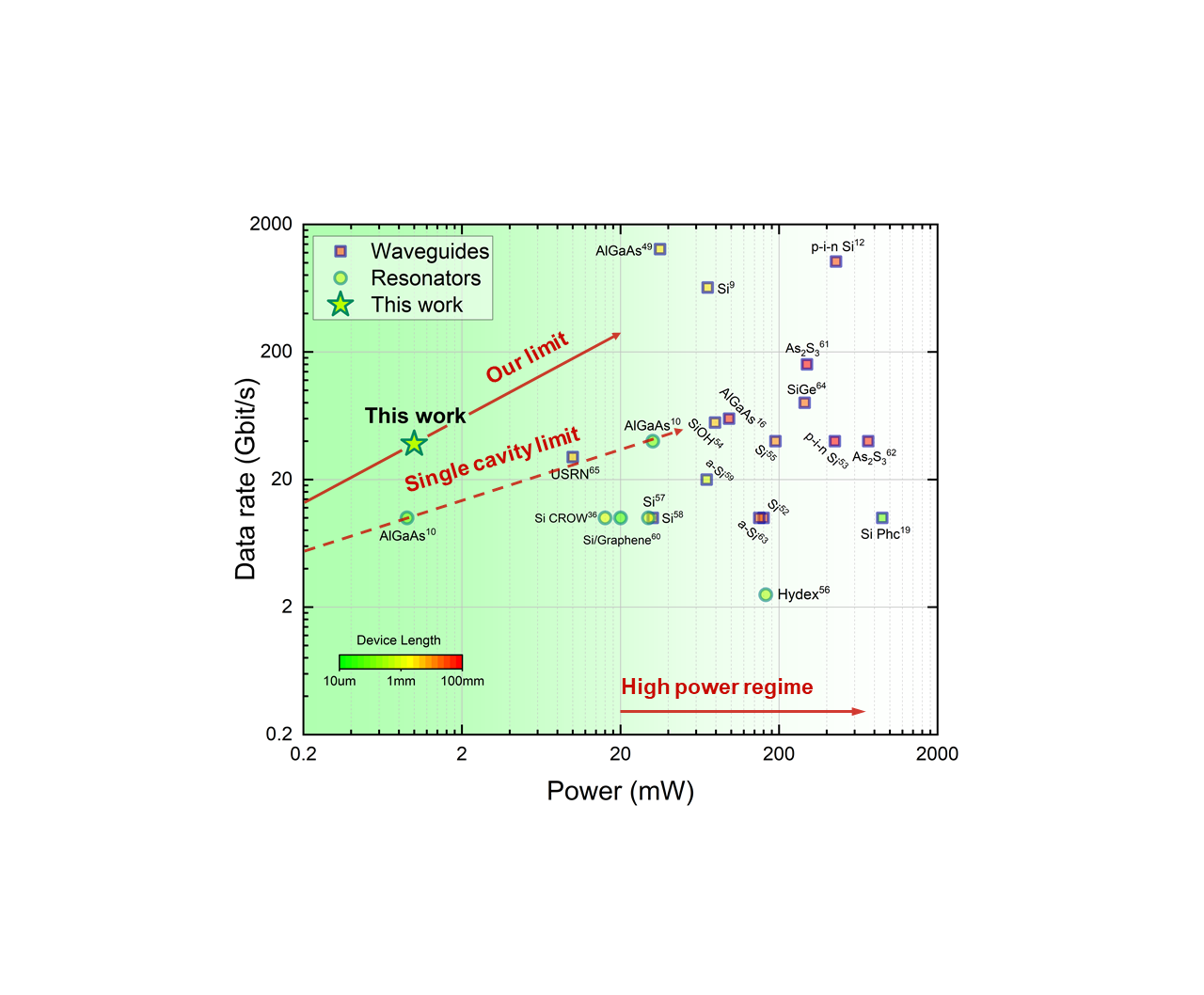
**

**Extended Data Fig. S1 | Performance comparison of reported all-optical wavelength conversion systems realized on integrated photonic platform.** Rectangular, circular, star-shaped markers represent the type of the reported devices: waveguides, resonators, and this work, respectively. The color shading of the markers indicates the device lengths.


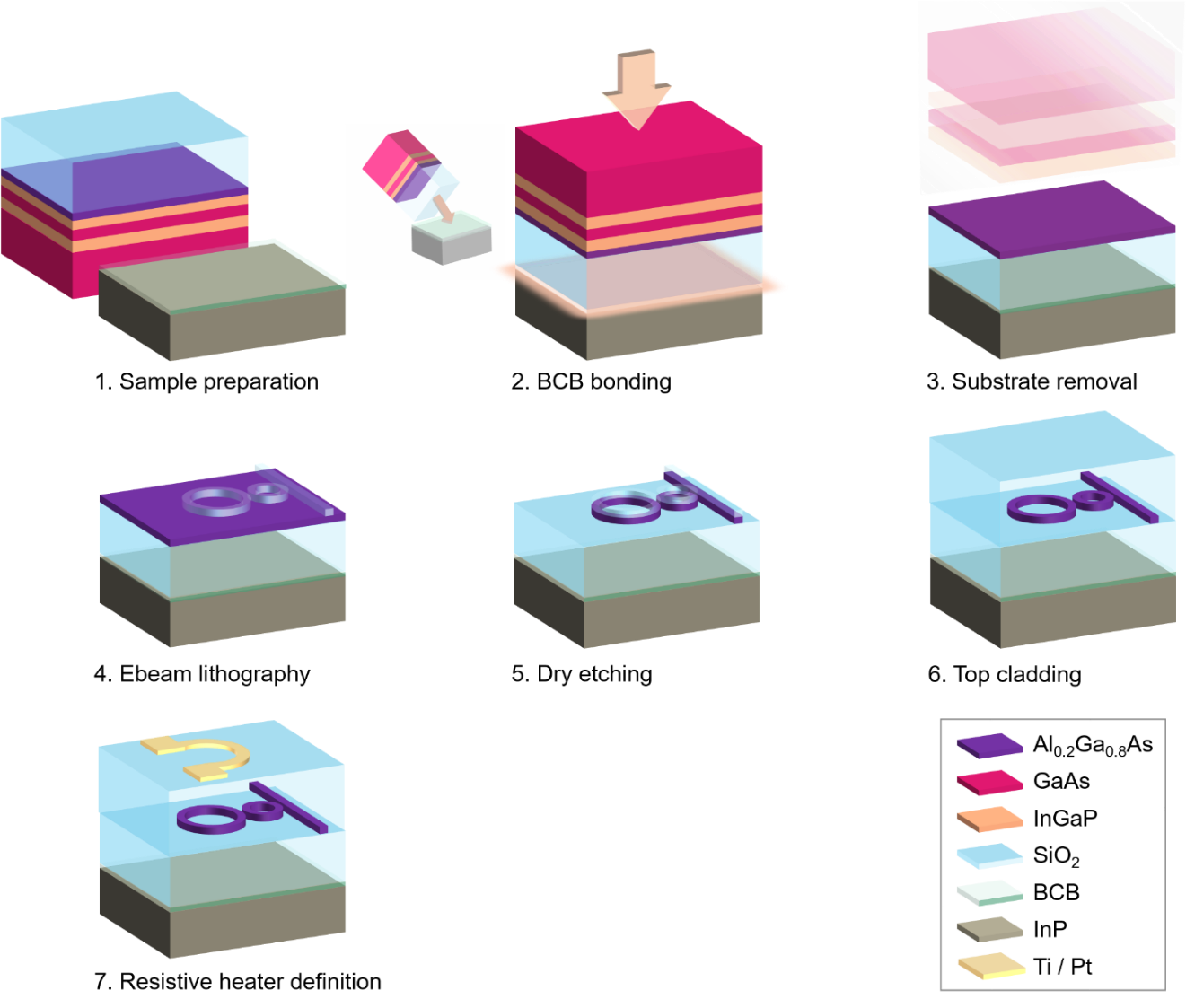


**Extended Data Fig. S2 | Fabrication process of coupled resonator devices.**


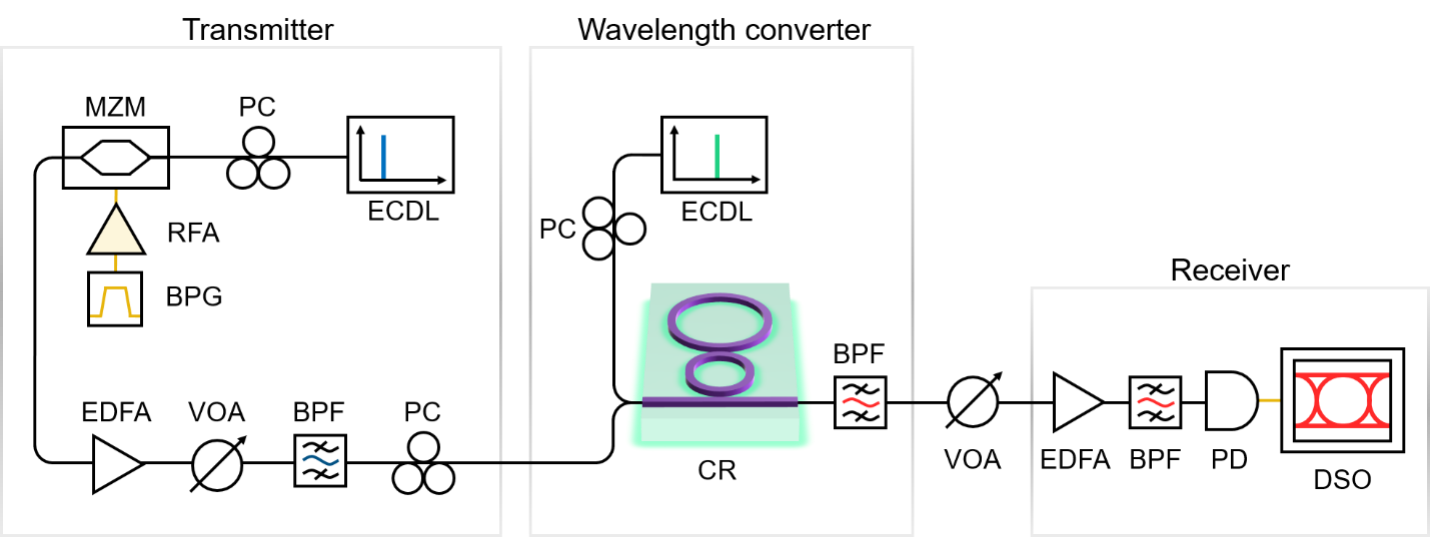


**Extended Data Fig. S3 | Schematic of the wavelength conversion experimental setup.** ECDL: external cavity diode laser, PC: polarization controller, MZM: Mach-Zehnder modulator, RFA: radio frequency power amplifier, BPG: bit pattern generator, EDFA: Erbium-doped fiber amplifier, VOA: variable optical attenuator, BPF: bandpass filter, CR: coupled resonator device, PD: photodiode, and DSO: digital storage oscilloscope.


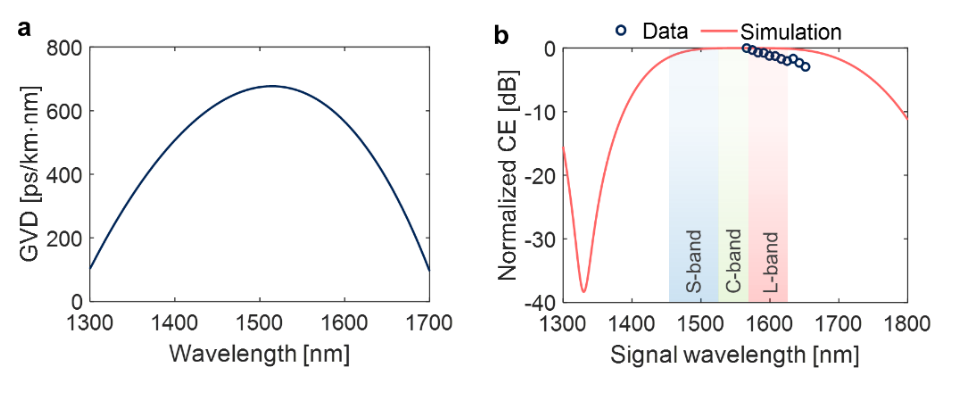


**Extended Data Fig. S4 | FWM wavelength conversion (phase-matching) bandwidth. a**, Simulated group velocity dispersion (GVD) of the AlGaAsOI waveguide with a width of 465 nm and a thickness of 290 nm. **b**, Red line is the simulated normalized conversion efficiency according to the GVD information in **a**, and blue circles are the conversion efficiency data points extracted from **Fig. 4d**.
